# Attention-based fusion of multiple graphheat networks for structural to functional brain mapping

**DOI:** 10.1038/s41598-023-50408-6

**Published:** 2024-01-12

**Authors:** Subba Reddy Oota, Archi Yadav, Arpita Dash, Raju S. Bapi, Avinash Sharma

**Affiliations:** 1 Computer Science, Inria Bordeaux, Bordeaux, France; 2 Cognitive Science Lab (CSL), International Institute of Information Technology (IIIT), Hyderabad, India; 3 CVIT Lab, International Institute of Information Technology (IIIT), Hyderabad, India

**Keywords:** Computational neuroscience, Network models, Computer science

## Abstract

Over the last decade, there has been growing interest in learning the mapping from structural connectivity (SC) to functional connectivity (FC) of the brain. The spontaneous fluctuations of the brain activity during the resting-state as captured by functional MRI (rsfMRI) contain rich non-stationary dynamics over a relatively fixed structural connectome. Among the modeling approaches, graph diffusion-based methods with single and multiple diffusion kernels approximating static or dynamic functional connectivity have shown promise in predicting the FC given the SC. However, these methods are computationally expensive, not scalable, and fail to capture the complex dynamics underlying the whole process. Recently, deep learning methods such as GraphHeat networks and graph diffusion have been shown to handle complex relational structures while preserving global information. In this paper, we propose a novel attention-based fusion of multiple GraphHeat networks (A-GHN) for mapping SC-FC. A-GHN enables us to model multiple heat kernel diffusion over the brain graph for approximating the complex *Reaction Diffusion* phenomenon. We argue that the proposed deep learning method overcomes the scalability and computational inefficiency issues but can still learn the SC-FC mapping successfully. Training and testing were done using the rsfMRI data of 1058 participants from the human connectome project (HCP), and the results establish the viability of the proposed model. On HCP data, we achieve a high Pearson correlation of 0.788 (Desikan-Killiany atlas with 87 regions) and 0.773 (AAL atlas with 86 regions). Furthermore, experiments demonstrate that A-GHN outperforms the existing methods in learning the complex nature of the structure-function relation of the human brain.

## Introduction

The human brain’s structural topology is estimated from diffusion tensor images (DTI) to derive the structural connectivity (SC) matrix that summarizes the fiber connectivity density among the brain regions. On the other hand, static or steady-state functional connectivity (FC) among these regions is estimated by computing the correlation coefficient (usually Pearson) of the respective time-varying resting-state functional magnetic resonance imaging (rsfMRI) signals. The correlation captures the spontaneous brain activity when participants are not engaged in any specified task. Furthermore, the brain activity observed in the rsfMRI signals is constrained and influenced by the connectome^1,2^. Characterizing the SC-FC mapping is an open and challenging research problem in cognitive neuroscience^3^. Such models can be used to identify the biomarkers that underlie any deviation from the expected FC based on the SC in various diseases such as Autism Spectrum Disorder (ASD), Dementia, and many more^4,5^. These models will also be helpful in characterizing the functional recovery patterns resulting from therapy by comparing the FC observed with the predicted FC based on healthy structural topology^4,5^.

Traditionally, graph-based modeling has been popular for solving SC-FC mapping. One of the seminal works in this direction by Abdelnour et al.^3^ formulated a linear model that considers the diffusion of regional brain activity over the graph topology by choosing a single optimal diffusion kernel. Later^6,7^, utilized multiple diffusion kernels for learning the SC-FC mapping and demonstrated the superiority of using multiple diffusion kernels. The idea of multiple diffusion kernels formulation is specifically interesting and demonstrates the integration of multiple kernels within the same machine learning model. Becker et al.^8^ proposed a versatile nonlinear mapping approach to obtain the functional connectivity from the structural connectivity random walks using spectral graph theory. In another relevant work on functional brain connectivity, [5] derived a relation between SC and FC via Laplacian spectra, where FC and SC share eigenvectors and their eigenvalues are exponentially related. However, most of these methods suffer from the computational overhead related to scalability or exhibit sub-optimal performance on SC-FC mapping over brain graphs.

A novel deep learning method called graph convolution network(GCN)^9^ has recently been proposed to generalize convolutional neural network models for graph data. GCNs achieve state-of-the-art results in various application domains such as, computer vision^10^, applied chemistry^11^, natural language processing^12^, and neuroscience^13^. The GCN-based Encoder-Decoder network was proposed for SC-FC mapping where the normalized Laplacian of SC was provided as input to GCN, and training was accomplished using the ground truth FC with an MSE loss function^14,15^. The primary limitation of the GCN-based Encoder-Decoder method is the absence of diffusion over multiple scales that is useful for the integration of information from the node attributes and the network topology. A recent variant of GCN, *GraphHeat Network* (GHN), attempts semi-supervised classification^16^ and enables control over heat diffusion scales while filtering out the influence of high-frequency spectral components of the graph Laplacian. Another recent work proposed by^17^ learned the graph kernels based on the intuition of the specific application domain instead of choosing standard kernels such as heat kernels and normalized heat kernels. However, the reported performance is poor on the SC-FC mapping experiments. Deep learning models such as CNNs and LSTMs, including GCNs, require large datasets for training and evaluation. Hence, in this paper, we evaluated our A-GHN model on the HCP dataset (i.e. 1058 SC-FC pairs) to learn the complex FC structure.

Recently, attention mechanisms have become popular and standard to enable working with variable size inputs and for focusing on the most relevant parts of the input to make decisions^18,19^. In the proposed model, A-GHN incorporates the propagation and aggregation of node representations by heat diffusion mechanism at multiple scales over the SC matrices. It is expected that the multiple scales contribute differently to the predicted FC. Thus, we introduce the attention mechanism to capture the contribution of each scale-specific A-GHN sub-models for learning the SC-FC mapping.

**Figure 1:**
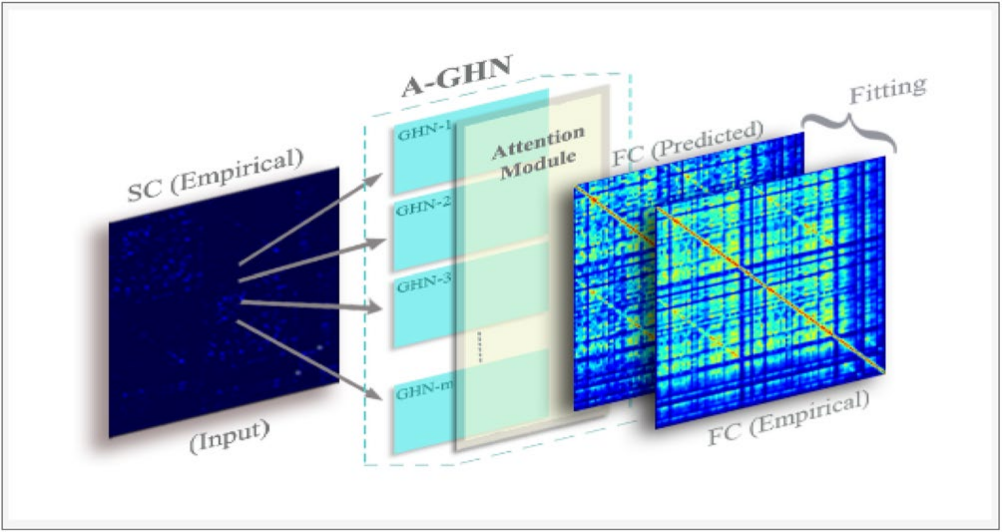
Mapping the structural and functional connectivity in brain graphs using the proposed A-GHN network.

In summary, these methods together establish the usefulness of single or multi-scale diffusion^3,7^ and the feasibility of graph neural networks for solving the SC-FC mapping problem^14^. Inspired by these and the attention mechanism over multiple scale-specific GHNs, we propose an attention-based fusion of multiple GraphHeat networks (A-GHN) that efficiently employs multi-scale diffusion to promote computational tractability and scalability. Figure 1 display the pipeline of A-GHN model. The A-GHN model utilizes multiple GHNs, each with an independent channel of input based on heat kernels. Predicted FC is then computed based on the weighted combination of these outputs and is compared with the empirical FC. Here, the attention scores are computed by taking the softmax over weight coefficients, where each attention score corresponds to the A-GHN sub-models output. As a result, the proposed model approximates the empirical FC well, and the FCs recovered with the A-GHN approach seem to have better correspondence with the ground truth than related models that incorporate either multi-scale diffusion or GHN. The key contributions of this paper are the following:

- We propose a novel, end-to-end learnable A-GHN architecture for learning the SC-FC mapping on brain graphs.
- Our method is grounded in the theory of the reaction-diffusion process in the cognitive domain while retaining the key properties of generalizability, scalability, and tractability in the deep learning framework.
- We present a comprehensive empirical analysis, including perturbation experiments and a detailed ablation study, to demonstrate the proposed model’s robustness and validity on a large publicly available dataset.

## Related work

### Whole brain modeling of SC-FC

Classical methods proposed non-linear models of cortical activity, which were then extended to model whole-brain behavior via coupling between regions based on structural connectivity^20^. Also, the whole-brain computational models have been used as powerful tools to understand the relationship between structural and functional brain connectivity by linking brain function with its physiological underpinnings^21–23^. Several other studies place non-linear oscillators at each cortical location and likewise couple them using anatomic connectivity strength^24–26^. However, these simulation models are only revealed through large scale, fine-grained stochastic simulations over thousands of time samples, and pose a practical challenge for the task of inferring functional connectivity from structural connectivity.

**Figure 2:**
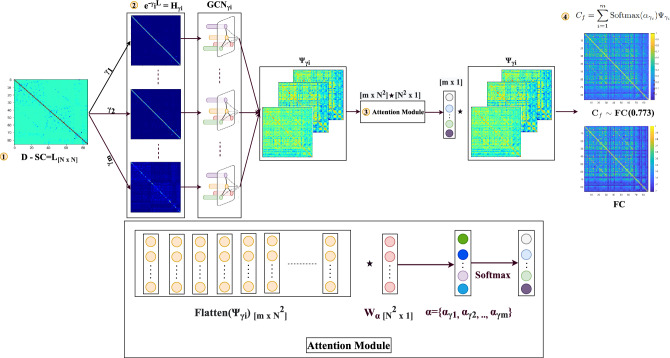
Proposed A-GHN architecture for learning SC-FC mapping using multi-scale GraphHeat networks (GHN) along with attention mechanism. A Laplacian matrix is computed from the structural connectivity matrix (SC) input in step 1. Multiple heat kernel matrices are obtained using m different diffusion scales and fed to the individual (A-GHN sub-model) in step 2. In step 3, an attention module is introduced to learn the attention scores corresponding to A-GHN sub-models. A Softmax linear combination of the outputs $\Psi _{\gamma _i}$ yields the predicted functional connectivity ($C_f$), which is compared with the ground truth empirical FC in step 4.

### Graph-theoretic modeling using linear models

The earlier graph-theoretic modeling experiments studied the mapping of SC-FC relationships by capturing the correlation structure of whole-brain dynamics using linear models^3^. Specifically^3^, present a simple, low-dimensional network diffusion linear model producing an accurate description of the SC-FC relationship. However, this model uses one global parameter across all the subjects, and the hypothesis of a single scale best-fitting kernel across subjects is not tenable. Surampudi et al.^6^ observed that the combination of multiple diffusion scales exhibits scale-dependent relationships among various regions of interest (ROIs), and these multi-scale diffusion kernels can capture reaction-diffusion systems operating on a fixed underlying connectome (SC). However, multiple diffusion kernels were not sufficient to explain the self-organizing resting-state patterns found in FC. Recently, a new framework, the multiple kernel learning model (MKL), provides plausible mathematical reasoning for the existence of these co-activations along with diffusion kernels by linearizing a variant of the reaction-diffusion model and extending it to generate FC^7^.

### Deep learning models for SC-FC mapping

The earlier deep learning modeling experiments studied the SC-FC mapping using an autoencoder (multi-layer perceptron as neural network architecture) method^27^. Recently, the study of GCNs has successfully reconstructed the brain FC from an SC graph by building a graph encoder-decoder system^14^. Moreover, the learned low-dimensional embeddings capture essential information regarding the relationship between functional and structural networks. In another recent work^15^, investigated the SC and FC mapping within a deep learning GNNs-based framework, including graph convolutional networks (GCN) and graph transformer networks (GTN). However, the major limitation of these methods is that they have not utilized either single diffusion kernel at an optimum scale or multiple scales of diffusion. A recent work in these lines^17^ proposed a deep graph spectral evaluation network (GSEN) for modeling the graph topology evolution by the composition of a newly generalized kernel. This method efficiently models the global and local evolution patterns between the source (SC) and target (FC) graphs. Global patterns involve features that reflect the general characteristics or relationships present throughout the entire graph structure, whereas local patterns capture the finer details of interactions, considering the influence of nearby nodes or connections within a limited vicinity. Although the method seems interesting, the GSEN model reports a poor performance on SC-FC mapping.

## Proposed solution

### Problem statement and proposed solution

The brain is typically represented as a graph in the computational neuroscience community, where graph nodes are modeled as key brain regions, and edges represent their structural or functional relationships. The aim here is to learn a mapping between the two brain graphs representing a sparse structural connectivity matrix (SC) and a dense static (steady-state) functional connectivity (FC) matrix, as depicted in Fig. 2. We propose to employ multi-scale heat diffusion kernels in a novel deep learning framework for this task.

### Mathematical background and notations

#### Graph definition

Consider a weighted, undirected graph denoted by ${\mathcal {G}} : = ({\mathcal {V}}, \textit{A}, \textit{E}$), where ${\mathcal {V}}$ is a set of *N* nodes, *A* $\in {\mathbb {R}}^{NXN}$ is the symmetric adjacency matrix and *E* is the set of edges connecting the nodes. A graph Laplacian matrix is defined as *L* = *D* – *A*, where *D* is a diagonal matrix with degree of nodes on the diagonal, $D_{i,i} = \sum _{j}{} \textit{A}_{ij}$ The spectral decomposition of the Laplacian matrix ($\textit{L} = \textit{U}\Lambda \textit{U}^T$) yields (i) Eigenvector matrix (*U*) and (ii) Eigenvalue matrix ($\Lambda$) which is a diagonal matrix with the eigenvalues arranged in increasing order.

#### Graph convolutional networks

Graph Convolutional Neural network (GCN) is a multi-layer neural network that convolves neighboring node’s features and propagates a node’s embedding vectors to its nearest neighborhood^9^. For a one-layer GCN with Z hidden units, the latent node feature representation ($\Psi ^{(1)}$) is computed as

1 $$\begin{aligned} \Psi ^{(1)} = f(AXW_{0}) \end{aligned}$$

where *A* is the symmetric adjacency matrix, $\textit{X} \in {\mathbb {R}}^{NXM}$ is the node feature matrix where each row of the matrix represents a M-dimensional content vector for each node in the graph, $W_{0} \in {\mathbb {R}}^{MXZ}$ is weight parameter associated with the $1^{st}$ layer of GCN, and *f* is activation function. One can incorporate higher-order information of the neighborhoods by stacking multiple GCN layers

2 $$\begin{aligned} \Psi ^{(i+1)} = f(A\Psi ^{(i)}W_{i}) \end{aligned}$$

where *i* denotes layer number and $\Psi ^{0}=X$

#### Graph convolution using heat kernel

The *GraphHeat Network* (GHN) formulation captures the smoothness of labels or features over the neighborhood of the nodes as determined by the graph structure^16^. A heat kernel is defined as

3 $$\begin{aligned} \rho (\lambda _{i}) = e^{-(\gamma \lambda _{i})} \end{aligned}$$

where $\gamma \ge 0$ is the scale hyper-parameter, and $\lambda _{i}$ denotes the $i^{th}$ eigenvalue in $\Lambda$. Let $\Lambda _\gamma = \rho (\Lambda ) = \text {diag}(\{\rho (\lambda _i)\}_{i=1}^{N})$ denote the kernelized diagonal matrix. Thus, we can define the convolution kernel ($g_{w}$) as

4 $$\begin{aligned} g_{w} = \sum _{k=0}^{K-1}w_{k}(\Lambda _{\gamma })^{k} \end{aligned}$$

where $w_{k}$ is the weight parameter and here we choose $\textit{K} = 2$ (*i.e.* only considering the first-order polynomial approximation of ChebyNet^28^).

For the given input signal *X*, graph convolution is achieved as follows:

5 $$\begin{aligned} \Psi _{\gamma }&= U g_{w} U^{T} X \nonumber \\&= U \left( \sum _{k=0}^{K-1}w_{k}(\Lambda _{\gamma })^{k}\right) U^{T} X \end{aligned}$$

Specifically, for our choice of *K* = 2:

6 $$\begin{aligned} \Psi _{\gamma }&= (w_{0}I_{N} + w_{1}e^{-(\Lambda _{\gamma })} UU^{T}) X \nonumber \\&= (w_{0}I_{N}+w_{1}e^{-(\gamma L)})X \nonumber \\&= (W_{\gamma }e^{-(\gamma L)})X \nonumber \\&= (W_{\gamma } H_{\gamma })X \end{aligned}$$

where $W_{\gamma }^{N \times N}$ is a weight matrix corresponding to scale $\gamma$, $H_{\gamma } = e^{-\gamma L}$ represents the heat kernel matrix, and $\Psi _{\gamma }^{N \times N}$ is the scale-specific output of GHN. Please note in the above equation that *X*, the node feature matrix, is taken as an identity matrix in this formulation. To reduce the number of free parameters and to avoid over-fitting, we assume $W_{\gamma }$ = $w_{0}$= $w_{1}$, and the equation becomes $W_{\gamma } (I_{N} + e^{-(\gamma L)} )X$. Therefore, adding the identity matrix enforces self-connections to the heat kernel matrix ($e^{-(\gamma L}$). However, our heat kernel matrix ($e^{-(\gamma L}$) already has self-connections; hence we ignored the identity matrix $I_{N}$. In the A-GHN formulation, multiple graphHeat models are considered for different positive scales; hence $w_{0}$ has been omitted as it does not affect the overall results.

### Attention based multiple graphheat networks (A-GHN)

Let us consider *m* heat kernel matrices with *m* different scales $\{\gamma _{1}, \gamma _{2}, \gamma _{3}, \cdots , \gamma _{m}\}$ and corresponding GraphHeat kernels $\{H_{\gamma _{1}}, H_{\gamma _{2}}, H_{\gamma _{3}},\cdots , H_{\gamma _{m}}\}$. A-GHN already includes the propagation and aggregation of node representations by heat diffusion mechanism over the SC matrix. Further, the weight matrix parameters associated with the structural graph are learned during the model training process, reflecting the mean regional activities. Hence, the node feature vector *X* was chosen as a one-hot vector (*I*$_{N}$) in our model setting.

Each A-GHN sub-model outputs a matrix $\Psi _{\gamma _{i}}$ and we hypothesize that the linear combination of the softmax probabilities with A-GHN sub-model outputs would give rise to a good estimate of FC. Let $\alpha = \{\alpha _{\gamma _{1}}, \alpha _{\gamma _{2}}, \cdots , \alpha _{\gamma _{m}}\}$ denote the weight coefficients in the linear combination corresponding to the *m* GHN branches (A-GHN sub-models). These weight coefficients are learned by feeding the outputs of all *m* GHN branches to a fully connected layer. In our proposed A-GHN model, the attention module is designed such that the differential contribution of multiple scales is weighted appropriately to estimate the predicted FC. In order to obtain the normalized weights (attention scores), we utilize the softmax activation function. Finally, the linear combination of the outputs of *m* GHNs weighted by the corresponding attention scores allows us to jointly train all A-GHN sub-models and the fully connected layer via end-to-end back-propagation learning.

7 $$\begin{aligned} \Psi _{\gamma _{i}}&= tanh (W_{\gamma _{i}}e^{-(\gamma _{i} L)}X) \end{aligned}$$

8 $$\begin{aligned}&= tanh (W_{\gamma _{i}}H_{\gamma _{i}}X), i \in [1, m]\end{aligned}$$

9 $$\begin{aligned} \alpha&= [vec(\Psi _{\gamma _{i}}), vec(\Psi _{\gamma _{i}}), \cdots , vec(\Psi _{\gamma _{i}})]_{m \times N^{2}} [W_{\alpha }]_{N^{2} \times 1} , i \in [1, m] \end{aligned}$$

10 $$\begin{aligned}&= [\alpha _{\gamma _{1}}, \alpha _{\gamma _{2}}, \cdots , \alpha _{\gamma _{m}}]_{[m \times 1]} \end{aligned}$$

where $\alpha _{\gamma _{i}} = vec(\Psi _{\gamma _{i}}) \times W_{\alpha }$ denote the linear coefficients capturing contribution of the individual heat kernel $\Psi _{\gamma _{i}}$.

Thus, we approximate the empirical FC with weighted combination of output of multiple A-GHN sub-models corresponding to *m* diffusion scales to predict the FC ($C_{f}$) as follows

11 $$\begin{aligned} C_{f} = \sum _{i=1}^{m}\text {Softmax}(\alpha _{\gamma _{i}})\Psi _{\gamma _{i}} \end{aligned}$$

#### Loss function

The attention parameters $W_{\alpha }$ and scale-specific parameters $W_{\gamma _{i}}$ are estimated from the training subjects (indexed by *s* that varies from 1 to *S*) and remain fixed during the testing phase. We consider the loss function *J* (Equation of 12) to be the mean squared error between empirical and predicted FCs. Since the target FC matrix is symmetric, we have also made the estimated FC matrix ($C_{f}$) symmetric by adding its transpose, similar to MKL^7^. The loss function is then minimized using the stochastic gradient descent procedure.

12 $$\begin{aligned} J&= \sum _{s=1}^{S}||C_{f}^{s} - \text {FC}^{s} ||_{F}^{2} \nonumber \\&= \sum _{s=1}^{S}||\sum _{i=1}^{m} \text {Softmax}(\alpha _{\gamma _{i}}^{s})\Psi _{\gamma _{i}}^{s} - \text {FC}^{s} ||_{F}^{2} \end{aligned}$$

    Here $\Psi _{\gamma _{i}}^{s}$ denotes a $N \times N$ matrix with subject index (s) and $\alpha$ denotes an attention $m \times 1$ vector. Figure 2 depicts the proposed architecture that combines attention-based fusion of A-GHN sub-models with multiple heat kernels.

### Relation to reaction diffusion phenomenon

Mutual interaction of the elements of a complex system results in a neural field of activity which in turn leads to the formation of self-organizing patterns. Reaction-Diffusion (RD) model is the mathematical framework that characterizes such a spatio-temporal change in the field. RD systems have been successfully used to model the interaction among neurons belonging to different brain regions and the associated functional connectivity (FC) among the regions of interest (ROIs) of the brain^29,30^. The reaction part of the RD model corresponds to the interaction of the excitatory and inhibitory neural elements, and the diffusion part corresponds to the spreading of the resultant neural activity over the structural fiber pathways. As the interacting (reacting) neural elements differ in their parameters, the emerging spontaneous activity of the neural ensemble results in non-linear patterns. The growth and the progression of a neural field are mathematically characterized by the Wilson-Cowan model, a variant of the RD framework. The statistical behavior of the mean activity of the neural fields is described by the equations of the Wilson-Cowan model^31,32^.

Inspired from the multiple kernel learning model (MKL) model^7^ which is based on the RD framework, in this paper, we propose attention-based multiple GraphHeat networks (A-GHN) to map SC-FC. The proposed solution formulation is analogous to MKL and is as follows:

#### MKL

The optimization formulation minimizes an objective function *J* comprising the mean squared error between empirical and predicted FCs as in^7^ and is represented as:

13 $$\begin{aligned} J&= \sum _{s=1}^{S}||\sum _{i=1}^{m}H_{i}^{s}\Pi _{i} - \text {FC}^{s} ||_{F}^{2} \end{aligned}$$

where $\Pi _{i}$ are estimated from the training subjects (indexed by *s* that varies from 1 to *S*), and $H_{i}^{s}$ denotes the Heat Kernel matrix of subject *s* associated with scale *i*.

Similarly, in^6^, the mixing coefficients are subsequently learned while solving an optimization formulation as:

14 $$\begin{aligned} {\hat{J}}&= \mathop {{\textrm{argmin}}}\limits _\alpha \sum _{s=1}^{S}|| \sum _{i=1}^{m}\alpha _{i}H_{i}^{s}- \text {FC}^{s} ||_{F}^{2} \end{aligned}$$

where $\alpha _{i}$ is a weight coefficient associated with scale specific heat kernel $H_{i}$

From Eqs. (7), (12), and (13), we observe that the learnable parameters ($W_{\gamma _{i}}^T$) in Eq. (12) in the proposed framework are analogous to the estimated parameters ($\Pi _{i}$) in Eq. (13) of the MKL framework^7^. Thus, as hypothesized in^7^, we can interpret ($W_{\gamma _{i}}^T$) as corresponding to the initial mean regional activities. Hence, $\Psi _{\gamma _{i}}$ in Eq. (12) of the proposed framework, when viewed along with Eq. (7), would correspond to the diffused output based on the initial mean regional activities.

Additionally, we introduce an attention mechanism in our proposed model (A-GHN) that combines attention scores with the outputs of *m* GHNs. From Eqs. (12) and (14), the learnable mixing coefficients through optimization formulation in Eq. (14) are analogous to the weighted attention scores obtained through gradient descent in Eq. (12).

We present the visualizations of ($W_{\gamma _{i}}$) and the correlation plot between the empirical and predicted FCs without attention in Section “Ablation studies”.

## Experimental setup and results

This section provides details of the experimental setup, dataset, model design, and comprehensive evaluation of the proposed model. Further, we performed detailed ablation studies where we induced perturbations in the input and conducted studies by removing the attention module to see the impact on the performance in all the cases and justify the proposed architecture.

### Dataset analysis

Deep learning models typically require a large amount of data for training as they involve learning a huge number of parameters. Further, MRI data acquisition comprising different modalities such as T1, DTI, and rsfMRI is a costly and time-consuming process. In light of these issues and in order to obtain a meaningful comparison against the existing results, we considered a popular and widely used dataset from the human connectome project (HCP) [ http://www.humanconnectomeproject.org/data/]. We have considered the structural connectivity - functional connectivity (SC-FC) pairs of a total of 1058 subjects from the HCP repository (see^33^ for data pre-processing methodology). All these participants underwent resting-state functional imaging (no task condition) with their eyes closed. The structural connectivity (SC) matrix, derived from diffusion tensor imaging (DTI), reveals the white-matter fiber connections between regions of interest (ROIs). The elements of the SC matrix correspond to the normalized count of streamlines connecting pairs of regions. On the other hand, the FC matrix is characterized by Pearson’s correlation of time series from resting state fMRI for different brain regions. The blood oxygen level-dependent (BOLD) time-series signal available for each participant has 1200 time points aggregated across 87 regions of interest (ROIs) as per the Desikan-Killiany brain atlas^34^. Therefore, 87 brain regions with 1200 time points result in $87 \times 87$ FC matrix. The HCP 1058 subjects dataset with Desikan-Killiany parcellation has been made available by *Zhang et al.*^35^ [https://github.com/maxwass/brain_data_processing]. We also evaluated our model on 100 subjects from the HCP repository as per the AAL brain atlas across 86 brain ROIs^36^. The HCP 100 subjects dataset with AAL parcellation is obtained from *Surampudi et al.*^37^.

### Baseline methods

Since the proposed model combines graph convolutional network with multiple heat kernel diffusion, we chose two related baseline methods for comparative analysis. The first method, multiple kernel learning (MKL) model proposed in^7^, utilizes multi-scale diffusion over brain graphs to learn the subject’s SC-FC mapping but does not incorporate deep networks. On the other hand, the second method uses GCN-based Encoder-Decoder architecture^14^ is a deep learning-based model. However, this does not incorporate multi-scale diffusion. Thus, the two baselines together allow us to evaluate the impact of deep networks and that of the multi-scale diffusion independently against our proposed A-GHN model. We replicated both the MKL and GCN Encoder-Decoder models with the same choice of parameters as indicated in the original papers on the data from 1058 participants from HCP for training and testing experiments. We further compared our A-GHN results with several previous state-of-the-art methods such as Autoencoder^27^, Macroscale mapping of SC-FC^38^, and Graph Neural Networks which uses both Graph Convolutional Network (GCN) and Graph Transformer Network (GTN)^15^.

### Model setup

Here, we describe the model setup, training and testing phases for the proposed A-GHN model.

#### Training phase

We trained the A-GHN model on HCP rsfMRI data where a randomly chosen set of 550 subjects of which 500 subjects used for training (500 SC-FC pairs), 50 subjects (50 SC-FC pairs) for validation and the remaining 508 subjects (508 SC-FC pairs) for testing. The 87 $\times$ 87 heat kernel matrix obtained from the Laplacian of structural connectivity (SC) matrix was given as input to the graph convolution networks (GCN) and the 87 $\times$ 87 empirical functional connectivity (FC) matrix as the target output to train the model. Here, the number of vertices corresponds to the 87 brain regions, and the edges represent the structural fibers connecting the brain regions over which heat diffusion takes place. As shown in Fig. 2, outputs of the one-layer A-GHN models were combined in a weighted manner using the corresponding attention scores obtained from the Softmax layer. The number of coefficients obtained is equal to the number of scales ($m=7$), and the final output is an ($87 \times 87$) predicted FC. We used mean squared error (MSE) between empirical and predicted FC matrices as the loss function for learning.

#### A-GHN hyper-parameters

To perform SC-FC mapping using A-GHN, we set the convolution layer’s embedding size as 87 and the input node feature vector *X* as the identity matrix ($\textit{I}_{N})$. We used Adam optimizer^39^ with an initial learning rate of 0.001, *tanh* as the activation function, and the $L_2$ weight decay was set to $5e^{-4}$. We applied dropout with a keep-probability of 0.5 and trained the A-GHN model for a maximum of 100 epochs. To overcome the over-fitting problem, we stopped training if the validation loss did not decrease for 10 consecutive epochs (See supplementary material for the profiles of learning curves in Fig. SF1).

**Figure 3:**
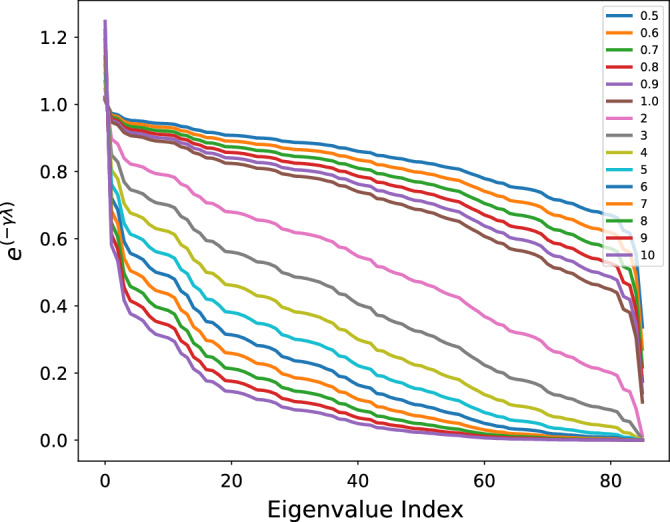
Depicts different diffusion scales ($\gamma$) ranging from 0.5-10 (values in the legend), and each exponential curve is a function of the scale ($\gamma$) and represents the contribution of every eigenvalue of the Laplacian of the SC matrix (the indices of eigenvalues (in increasing order) are shown on the abscissa).

#### Testing phase

**Figure 4:**
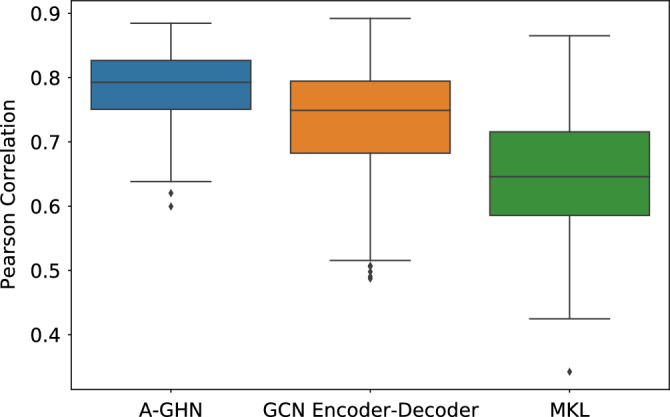
Pearson correlation values between empirical and predicted FCs of all the test subjects with the proposed A-GHN model (Green line), averaged over five runs, are compared with the predictions of the other two models. Horizontal lines show the mean correlation values (higher is better) of 0.788, 0.732, and 0.645, respectively, for A-GHN, GCN Encoder-Decoder, and MKL.

**Figure 5:**
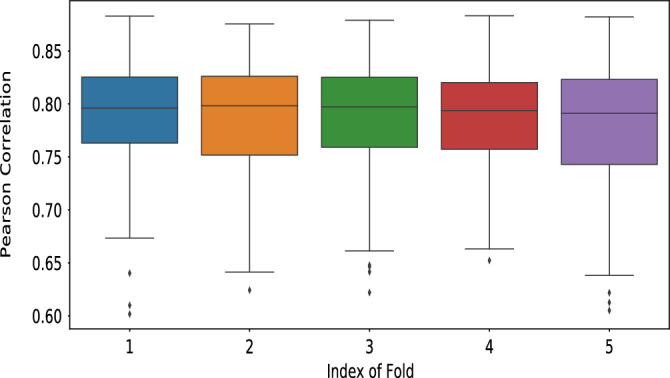
Results of performance of A-GHN model in the 5-fold cross-validation setting on 1058 subjects. The box plots depict the Pearson correlation between empirical and predicted FCs in each fold.

We used the other half (508 SC-FC pairs) to predict the corresponding FC matrices in model testing. We followed the same parameters used in model training except omitting the drop-out parameter. We use the Pearson correlation coefficient between the empirical (ground truth) and the predicted functional connectivity (FC) matrices to measure the model performance. There were two kinds of validation experiments performed—5-runs (each run with different random initialization), and 5-fold cross-validation (CV). For the 5-runs set-up, we report the average Pearson correlation over the 5-runs on the 508 test subjects. The average results for each test subject are shown in Fig. 4 (depicting Pearson Correlation values) and Fig. SF2 (depicting MSE values). For ease of visualization, we also report the results for randomly sampled 100 test subjects from the 508 test cohort in Figs. SF3 and SF4 (please refer to Supplementary material). For the 5-fold CV experiments, 4-folds are used for training and one-fold for testing. The results of the 5-fold CV are shown in Fig. 5. Thus, the validation results establish the generalizability of the results with different data splits.

Choice of Model Parameters The choice of various model parameters is explained below.

#### Choice of *m*

Figure 3 shows the profile of heat kernels for various scales of diffusion ($\gamma$) ranging from 0.5-10. The GraphHeat formalism^16^ allows for selective focus on low-frequency spectral components at higher scales, whereas high-frequency spectral components are suppressed at lower scales. Hence, in this paper, we chose multiple scales where each scale of diffusion characterise to determine neighboring nodes that reflect the local structure or the relevant information of smoothness manifested in the graph structure. As can be seen in Fig. 3, the local diffusion phenomenon is observed for smaller scales (0.5–1) with contribution from many eigenvalues/vectors, including the large eigenvalues. On the other hand, the global diffusion phenomenon is noticed for bigger scales (1-10) that depend predominantly on the contribution from eigenvalues/vectors corresponding to smaller eigenvalues. The number of heat diffusion scales (see Eq. 7) was set to $m=7$ empirically, based on the performance of the proposed model. We used ascending order of scales that correspond to the global diffusion phenomenon in case of lower scale indices ($\gamma$ values of 0.6 and 0.8) and local diffusion phenomenon in case of higher scale indices ($\gamma$ values of 1, 2, 4, 6, and 8) (see Fig. 3).

#### Choice of activation function

In order to determine the kind of activation function to be used in the output layer, we ran experiments with several choices and found that *tanh* is suitable. We observed that *tanh*, *relu*, and *leaky relu* (with a negative slope of 0.01) activation functions yielded similar performance values while the configuration with *sigmoid* function had a lower performance. Since the FC correlation matrix values are in the range of -1 to 1, we chosen *tanh* as the activation function in the output layer of the A-GHN for further experiments. These results are shown in Fig. SF7 in the supplementary material.

#### Choice of A-GHN layers

To understand the impact of increasing the number of hidden layers of A-GHN, we experimented with a two-layer, and four-layer A-GHN models. The empirical results show that the mean Pearson correlation of test subjects with the two-layer model (0.799) was marginally better than that of the one-layer model (0.788), as shown in Fig. SF8 (please refer supplementary). However, it appeared that a further increase in the number of layers (four layers) led to over-fitting and a decrease in performance (0.76). In order to estimate the statistical significance of the performance differences, we performed One-way ANOVA on the mean correlation values for the test participants across the A-GHN models with different depths. The main effect of model was significant [F(2,1506)=73.59, *p*=.0000]. Further, the *post hoc* pairwise tests revealed that the mean correlation values of the A-GHN model with one-layer was significantly different from those of the other two models [with two layers: *p*=.0.00004 and with four-layers: *p*=.0000]. Overall, as a trade-off we considered a one-layer A-GHN model for all further experiments based on its smaller training parameter-set.

**Table 1:** Comparison of A-GHN model with previous state-of-the-art models. Comparison is done by computing the Pearson correlation between the ground-truth FC and predicted FC of test subjects. Overall, the A-GHN model displays a higher correlation value of 0.788, better than previous models. Significant values are in bold.

Model	Correlation
MKL^7^	0.645
GCN Encoder Decoder^14^	0.732
Autoencoder^27^	0.561
Macroscale SC-FC^38^	0.501
GNN (GCN + GTN)^15^	0.715
M-GHN^40^	0.741
Random A-GHN (Freezing GHNs)	0.557
A-GHN (one-layer)	**0.788**
A-GHN (two-layer)	**0.799**
A-GHN (four-layer)	**0.760**

**Figure 6:**
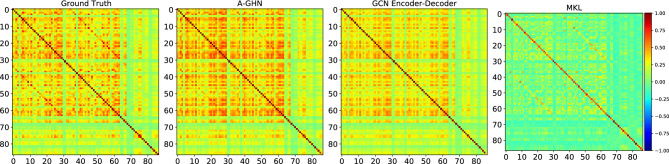
Qualitative comparison of the Functional Connectivity matrices (FCs). The mean of the predicted FCs from the proposed A-GHN model is compared with that of the mean FC from ground truth (empirically observed), GCN Encode-Decoder ^14^ and MKL ^7^ models.

**Figure 7:**
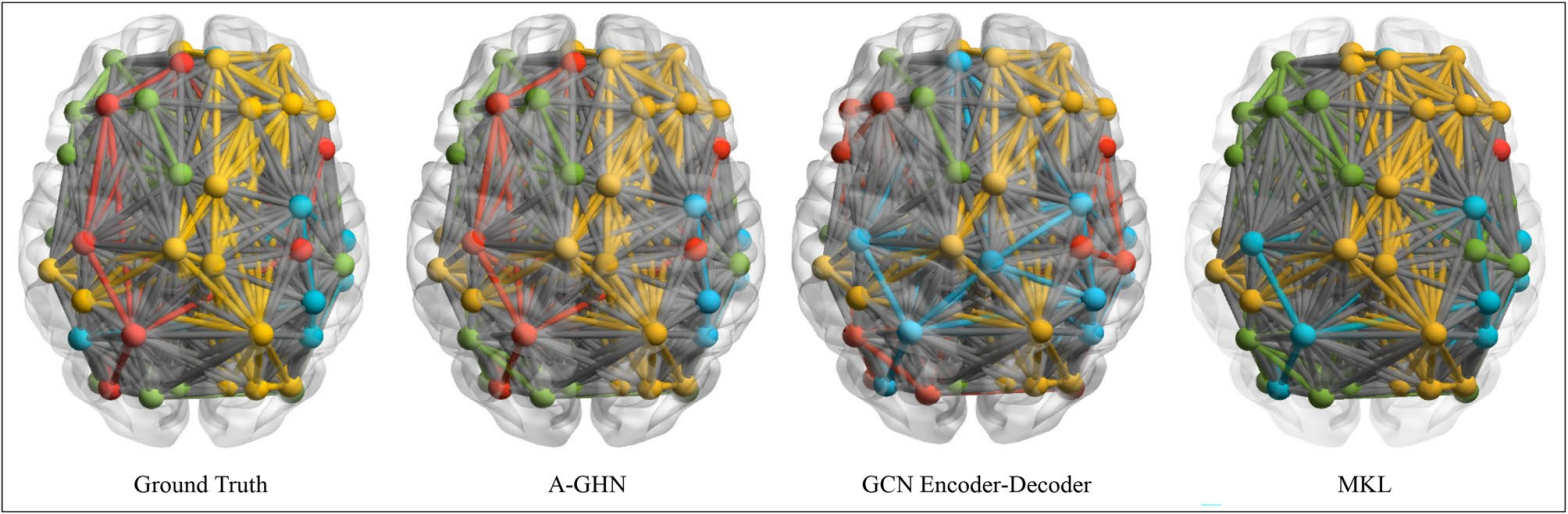
Qualitative comparison of the Functional Connectivity Networks. Four communities are derived from the mean FC matrices of the test subjects from the ground truth as well as the predicted FCs from the proposed and other models: MKL^7^ and GCN Encoder-Decoder^14^. Color coding of the edges/nodes for different models is done independently, and hence the cross-comparison of community structures is qualitative in nature.

### Results

#### Comparison with previous methods

Here, we compare the performance of the A-GHN model with baseline and existing models for the SC-FC mapping, as reported in Table 1. The comparison of the proposed A-GHN model is made across four groups of models: (a) general baseline models (Autoencoder, Macroscale SC-FC); (b) non-deep learning model but that uses multiple kernel diffusion (MKL); (c) GCN-based models (GCN Encoder Decoder, GNN); and (d) GraphHeat-based baselines (M-GHN, Random A-GHN). We make the following observations from Table 1: (i) On Pearson correlation, A-GHN is better across all the models. (ii) The results of the Random A-GHN model where all the GHN layers are kept frozen and untrained, yields a lower mean Pearson correlation than other GCN-based models. (iii) A-GHN with different layers shows superior performance as compared to multiple GHNs (M-GHN)^40^, Graph Neural Networks (combination of GCNs: Graph Convolutional Networks and GTNs: Graph Transformer Networks) and GCN encoder decoder based models. It is interesting to note that none of these models (except M-GHN) uses multiple scales of diffusion as in the proposed approach.

For further quantitative and qualitative analyses of A-GHN, we proceed with comparison against two of the above approaches: MKL and GCN Encoder Decoder models. While MKL model is a representative of multiple kernel diffusion strategy, GCN Encoder Decoder model signifies a typical graph-based deep learning approach.

#### Quantitative evaluation

We compared the performance of our proposed model with two existing approaches: Multiple Kernel Learning (*MKL*) model^7^ and the GCN-based Encoder-Decoder model^14^. The results of the comparative study using the 5-random-run experiments are shown in Fig. 4 & Table 1, where we can see that the proposed A-GHN model performs better with a mean correlation value of 0.788 in the range of [0.60, 0.885] on the test set as compared to GCN-based Encoder-Decoder model ($\hbox {Mean} = 0.732$, range in [0.487, 0.892]) and MKL ($\hbox {Mean} = 0.645$, range in [0.342, 0.865]). In order to estimate the statistical significance of the performance differences, we performed One-way ANOVA on the mean correlation values for the test participants across the three models. The main effect of model was significant [F(2,1506)=10.26, $p =.00007$]. Further, the *post hoc* pairwise tests revealed that the mean correlation values of the A-GHN model were significantly different from those of the other two models [with GCN Encoder-Decoder: $p =.03$ and with MKL: $p =.00004$]. On the other hand, the performance of the two baseline models did not differ significantly [GCN Encoder-Decoder vs. MKL: $p =.12$].

Similarly, Fig. SF2 (please see in the Supplementary) displays the mean squared error (MSE) of test subjects using the 5-random-run experiments, where the proposed A-GHN performs a lower MSE value of 0.0265 in the range of [0.013, 0.054] on the test set as compared to GCN-based Encoder-Decoder model ($\hbox {Mean} = 0.037$, range in [0.024, 0.067]) and MKL ($\hbox {Mean} = 0.086$, range in [0.015, 0.261]). Further, the statistical significance test using the one-way Anova test provides an F-statistic [F(2,1506) = 37.33, $p = 0$] concludes that the model was significant. Also, the post-hoc Tukey-HSD test reported that the proposed A-GHN model was significantly different with two models [with GCN Encoder-Decoder: $p =.016$ and with MKL: $p =.00001$].

Further, Fig. 5 depicts the results of 5-fold CV experiments, establishing the generalizability of the results with different data splits. From Fig. 5, we observe that A-GHN yield an equal performance across all the five folds. The box-plots in Fig. 5 depicts the range of Pearson correlation values across test subjects in that Fold.

#### Qualitative evaluation

We computed the mean of the predicted FC and the mean of the empirical FC matrices of the test subjects. We also computed the mean predicted FC matrices of the baseline models (GCN Encoder-Decoder and MKL). The visualizations of FC matrices are shown in Fig. 6. Here, we can observe a better qualitative match between the mean predicted FC of our proposed model and the mean ground truth.

In order to look at the finer details of the goodness of the learned mapping, four FC Networks were derived from the mean FC matrices of the test subjects using the Louvain algorithm available in the brain-connectivity-toolbox^41^. The edge-connectivity patterns of the predictions of the three models and the ground truth were rendered on a brain surface using BrainNet viewer^42^ to understand the similarity of node and edge distributions between the empirical and the predicted FCs, shown in Fig. 7. It can be seen that the proposed A-GHN model has a higher visual similarity to the empirical FC in terms of community assignment and inter-hemispheric connections as compared to the other models.

To empirically evaluate the community assignment across three models, we measure the mutual information based on the entropy (MI)^43^ between communities for ground truth FCs of the three models: A-GHN, GCN Encoder Decoder and MKL. The mutual information MI is computed as follows,

15 $$\begin{aligned} \begin{aligned} MI(X;Y) = D_{KL}(P_{(X,Y} \parallel P_X \otimes P_Y) \end{aligned} \end{aligned}$$

where X and Y are the two vectors (community assignments of the nodes as computed by the Louvain algorithm) in consideration, $D_{KL}$ is the Kullback-Leibler divergence. Since mutual information (MI) measures the similarity in the information captured between two communities, it can be seen as a global correspondence measure of the brain community structure^44^. The higher the MI, the more the similarity with the ground-truth FC. Table 2 reports the mutual information between different pairs of community assignments for the predicted FCs in the three models with the communities detected in the Ground truth FC. The community detection was done on the average of all the 508 test subjects. It can be observed from Table 2 that A-GHN model has higher mutual information with ground truth FCs (1.357) across all the test subjects compared to GCN Encoder-Decoder (0.735) and MKL (0.740).

**Table 2:** Mutual information between the communities detected in various models. Comparison is done by computing the mutual information (MI) between the four communities across different models. Overall, the A-GHN model has higher mutual information with Ground Truth.

Model1 vs. Model2	Mutual Information
Actual vs. Actual	1.706
Actual vs. A-GHN	**1.357**
Actual vs. GCN Encoder Decoder	0.735
Actual vs. MKL	0.740
A-GHN vs. A-GHN	2.180
A-GHN vs. GCN Encoder Decoder	0.847
A-GHN vs. MKL	1.064
GCN Encoder Decoder vs. MKL	0.675

### Ablation studies

We performed various ablation studies to establish the robustness of the proposed model. As an initial step, we estimate the native correlation between the ground truth SC & FC and compare with the SC-FC correlation obtained for the test data using the proposed A-GHN model. Subsequently, an ablation study was carried out to measure the importance of the attention module which is a key element incorporated in the proposed model. The relation between the size of the training data set and the model performance has also been studied. We have also conducted additional perturbation studies to verify whether our model learns the SC-FC relationship correctly and does not simply over-fit the data. One experiment studies the impact of perturbing the test input when the training protocol is intact. The second one verifies the results when the model was trained using perturbed inputs but tested on the original target outputs.

#### Comparison with the native SC-FC correlation

As an initial evaluation strategy, we report how the SC-FC correlations between the ground truth compare against those that are predicted from the 508 subjects’ test data using the proposed A-GHN model. From the results shown in Fig. SF1 (please see in the supplementary), we observe that the mean correlation between SC vs. FC-Actual (-0.0065) is comparable with the SC vs. FC-Predicted (-0.0024) for the proposed A-GHN model.

#### Importance of attention

The distinguishing feature of the proposed A-GHN model is the use of attention in order to estimate a weighted combination of the GHN outputs. In order to assess the importance of the attention module, we performed an ablation study. The model was run without attention (called M-GHN^40^ in Fig. SF12 (please see in the Supplementary) weights by simply summing and averaging the outputs of the seven A-GHN sub-models to obtain the predicted FC. It can be observed in Fig. SF12 that attention makes a difference in that the mean correlation value of A-GHN is 0.788 [range: (0.60, 0.885)] as compared to 0.741 [range: (0.461, 0.873)] of M-GHN. An F-test establishes that these differences are statistically significant [F(1, 1014)=5.3427, *p*=.023]. Similarly, we report the mean squared error (MSE) of test subjects using both A-GHN and M-GHN models in Fig. SF5. From Fig. SF5, we can observe that the overall MSE value of A-GHN is 0.0254 low as compared to 0.0302 for M-GHN.

#### Perturbation experiments with testing dataset

We perturbed the data corresponding to the 508 test subjects from the 5-run experiment reported earlier, where each subject was perturbed $N=250$ times. Here, each test SC matrix was perturbed by randomly generating the values of the elements from a power-law distribution that the elements are known to follow^45^. The A-GHN model was trained on unperturbed data of SC-FC pairs (550 subjects), and the resulting model was tested on each perturbed set of the test SC-FC pair. Figure SF14 (Please see in the supplementary) depicts the distribution of average Pearson correlation scores for these experiments. It can be observed that the model learned from the 550 unperturbed SCs performs rather poorly in predicting the FCs estimated from the randomly generated SCs. The histogram of mean correlation values ranges in [0.12, 0.45] with a mean correlation around 0.3, thus indicating that the model performance deteriorates when fed with random structural connectivity information during the testing period. Thus, we can empirically conclude that the proposed model indeed learns SC-FC mapping, and the FC predictions are not independent of SC but respect the topology/structure of the input.

We reported other ablation studies experiments such as (i) Random A-GHN (Fig. SF9), (ii) Varying the Training Data Size (Fig. SF10), (iii) Perturbing the Model Input (Fig. SF15), and (iv) Leave-One-Out Results on 100 HCP subjects with AAL Atlas (Fig. SF16), in the supplementary.

## Discussion

The study of the relationship between structural connectivity and functional connectivity and how the functional activity of the brain is generated from the anatomical structure has been a major research topic in the field of cognitive neuroscience. Several methods have been proposed to explore the mapping between SC-FC including, whole brain computational models^21,22^, simple linear diffusion models^3^ as well as complex non-linear models^24,26^, and linear multi-scale diffusion models^6,7^. The whole brain computational models have been used as powerful tools to understand the relationship between structural and functional brain connectivity by linking brain function with its physiological underpinnings. On the other hand, non-linear complex drift-diffusion models based on excitatory and inhibitory neuronal populations, though not analytically tractable, give rise to rich dynamics. Abdelnour et al.^3^ introduced a graph-based model with a linear single scale diffusion kernel at an optimal scale over the structural graph topology (SC) to map FC. However, Surampudi et al.^6^ showed that single kernel models do not generalize to a larger cohort and demonstrated that FC can be decomposed into multiple diffusion kernels with subject non-specific combination coefficients. Further, the MKL framework, proposed by Surampudi et al.^7^, revealed that the combination of multiple diffusion kernels was not sufficient to explain the self-organizing resting-state patterns found in FC and hence necessitated the use of additional explanatory parameters.

In this paper, we adopt the representation of the graph signal in terms of graphheat kernel similar to GraphHeat proposed by^16^. The GraphHeat formalism allows for selective focus on low-frequency spectral components at higher scales, whereas high-frequency spectral components are suppressed at lower scales. We consider a bank of such GHN models, each associated with a scale-specific heat kernel over the SC graph as input. The proposed A-GHN model then combines the outputs of the scale-specific GHN models using attention-based fusion. Both the hidden parameters ($W_{\gamma _{i}}$) associated with the scale-specific GHN models as well as the attention scores that combine the A-GHN sub-model outputs are jointly learned to estimate the empirical FC accurately. We have established a correspondence between the initial regional co-activation parameters ($W_{\gamma _{i}}$) in the proposed model and the parameters ($\Pi _{i}$) from the MKL framework^7^. It is to be noted that the MKL framework is shown to be a variant of a reaction-diffusion system on the graph topology determined by the underlying structural connectivity (SC) matrix. Thus, the proposed A-GHN method is grounded in the theory of the reaction-diffusion process in the cognitive domain.

The proposed A-GHN model displays superior performance as compared to baseline models such as GCN Encoder-Decoder^14^ and MKL model^7^. The model is able to learn population patterns regarding the SC-FC relationship even with smaller datasets. We validated our proposed model in two different settings: (i) 5-runs with the random initialization, and (ii) 5-Fold cross-validation. The experimental results showed that the correlation structure of the BOLD functional resting-state brain networks is significantly well captured by our model (Fig. 4). The predicted mean correlation for 508 test subjects is close to 0.788 (5-Runs experiment), whereas the GCN Encoder-Decoder and MKL yield (0.73), and (0.645), respectively. We conducted several ablation studies and perturbation experiments to establish the robustness of the reported results.

As explained below, the proposed framework enjoys three key properties of generalizability, scalability, and tractability in the deep learning framework.

### Interpretability and generalizability

We formulate the deep learning model, A-GHN, as an end-to-end framework for SC-FC prediction. The challenge in applying deep learning models to neuroimaging research lies in the black-box nature of the process, where it is hard to decipher what the deep network actually learns. In order to address this and to understand the model mechanisms, we devised the following: (i) deciphering the learned parameters $W_{\gamma _{i}}$, (ii) visualising the outputs of *m* number of A-GHN sub-models ($\Psi _{\gamma _{i}}$), and (iii) displaying the heatmap of attention probabilities across the test subjects (508 pairs of SC-FC), as shown in Figs. SF18, SF19, and SF20, respectively (Please see in the Supplementary).

**Table 3:** Comparison of A-GHN scale outputs with Ground Truth communities. Mutual information between the communities detected in various scales of A-GHN model. Comparison is done by computing the mutual information (MI) between the four communities across different scales. Overall, A-GHN model has higher mutual information with Ground Truth.

Model1 vs. Model2	Mutual Information
Actual vs. Actual	1.706
Actual vs. A-GHN Scale-0.6	0.841
Actual vs. A-GHN Scale-0.8	1.189
Actual vs. A-GHN Scale-1	1.312
Actual vs. A-GHN Scale-2	0.971
Actual vs. A-GHN Scale-4	0.589
Actual vs. A-GHN Scale-6	0.546
Actual vs. A-GHN Scale-8	0.443

From Figs. SF18 and SF19, we observe that lower scales display mean regional activity local to the neighboring nodes by suppressing the high-frequency spectral components. However, as the scale value increases, the large neighborhoods are taken into account with a global structure and captures much more information while discarding some irrelevant low-order neighbors. Thus, the proposed A-GHN model thereby be tuned to produce both local and global connectivity at lower and higher scales, respectively. Similarly, Fig. SF20 reports that the contribution of attention probabilities is decreasing as the scale value increases. Further, we performed community detection to identify the different networks captured in the FC predicted by the model. The communities were detected using the Louvain algorithm as described in the Brain Connectivity Toolbox (BCT)^41^. From Fig. SF21, it is observed that the communities detected in the predicted FC when compared with empirical FC (ground truth), capture the inter-hemispheric patterns very well.

Similar to the mutual information analysis done for the communities across various models, we perform mutual information between the scales and ground truth based on Eq. (15), where X and Y represent the communities detected in each scale-specific output of the A-GHN model and the ground truth, respectively. Table 3 shows the comparison of ground-truth similarities captured in the scale outputs. Scale-1 is the most similar to the ground truth in terms of its modularity and detected communities.

### Scalability and computational efficiency

The results reported in the current work use the parcellation based on the Desikan-Killiany Atlas ($87 \times 87$). We also report our A-GHN model results on 100 HCP subjects with AAL parcellation ($86 \times 86$), as shown in Fig. SF17 (please refer Supplementary). Nevertheless, the A-GHN model is easily scalable to any brain parcellation (for example, Gordon Atlas with $333 \times 333$, or Glasser Atlas with $360 \times 360$ parcellations). Graph-based diffusion models^3,7,37,46^ are not easily scalable for larger parcellations as the matrix operations are difficult to scale for larger matrix sizes. On the other hand, since graph convolutional network (GCN)-based models^14^ including the proposed A-GHN model use only node aggregate features that require vector operations; they are easily scalable.

From a computational efficiency perspective, one of the major limitations of the MKL model^7^ is that it uses LASSO optimization that requires computationally expensive matrix inverse operations. Hence the computational complexity is dominated by the cost of LASSO optimization. In contrast, the proposed A-GHN model is more efficient as it uses a stochastic gradient-based backpropagation learning approach. Moreover, the A-GHN model requires learning of 60,552 parameters (7 scales: 7x7569 + Attention Module: 1x7569) that is comparatively lower than learning 118,336 parameters in the MKL framework (16 scales: 16x7569). Further, the proposed framework is inherently scalable to more diffusion scales, more hidden layers in the GHNs, and can potentially be used for transfer learning on other datasets—all these make the proposed A-GHN model very flexible and computationally powerful.

### Limitations and future work

Usually, deep learning models require large datasets to obtain reliable learning and generalization performance results. An interesting point to note of our work is that it is trained and tested on a medium-size dataset of 1058 participants’ data. We demonstrated how A-GHN can be trained to obtain superior results using hyperparameter tuning and various validation experiments even with such a dataset. It would be interesting to demonstrate how A-GHN scales to larger datasets in the future. This research is the first step in applying the A-GHN model to perform automatic resting-state FC prediction from SC. In the near future, we intend to use the A-GHN model as a universal model to predict the FC of different types (both resting-state FC as well as task-based FCs) with the structural graph given as input.

In future work, a biophysical interpretation of the proposed deep learning model (A-GHN) with multi-scale heat kernel diffusion as an instance of a reaction-diffusion system on the structural brain graph needs to be established. Additionally, the proposed model could be used to characterize disease groups as well. It is to be notes that the proposed A-GHN considers average functional connectivity, ignoring the transient functional dynamics over the period of acquisition of the temporally extended rsfMRI signal. The proposed framework could potentially be extended to capture the temporal information in the functional connectivity dynamics (FCD). Finally, the current results utilize the well-known Deskian-Killiany (D-K) atlas that is representative and that has been used in many studies. However, in future we should look at other atlases such as Power2000, Brainnetome, etc.

## Conclusion

This paper proposed a novel A-GHN model that outperforms existing models that use either multiple diffusion kernels (MKL) or that use GCNs (GCN Encoder-Decoder). The current work demonstrates the feasibility of the A-GHN model with experiments on a large-size dataset of 1058 participants. Extensive cross-validation, perturbation, and ablation studies establish the robustness of the proposed architecture for learning the structure-to-function mapping of the brain using the images from DTI and rsfMRI. The model not only captures the SC-FC mapping but the underlying functional connectivity networks as well. The strengths of the deep learning based GHN models over graph diffusion-based linear models such as the MKL model are their computational efficiency and scalability.

### Supplementary Information

Supplementary Information.

## Data Availability

All data generated or analysed during this study are included in this published article *Zhang et al.*^35^. We did not create any new data as part of this work. We used the HCP dataset which is publicly available without any restrictions. HCP dataset can be downloaded from brain_data_processing github repository https://github.com/maxwass/brain_data_processing
