# Supplementary material for: Attention-based fusion of multiple graphheat networks for structural to functional brain mapping
Source: Sci Rep. 2024 Jan 12;14:1184. doi: 10.1038/s41598-023-50408-6 (PMC10786906; doi:10.1038/s41598-023-50408-6)
Supplement: Supplementary file 1 — Supplementary Information. [file 41598_2023_50408_MOESM1_ESM.pdf]

1 Supplementary Results

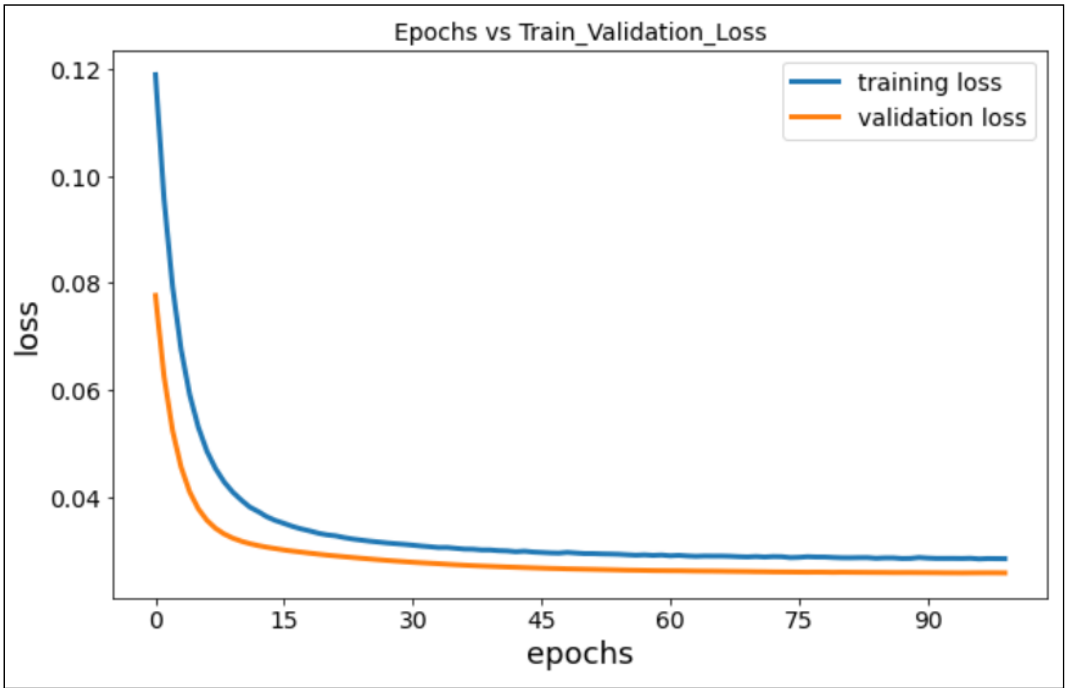

Figure SF1. Comparison of A-GHN performance on train/validation loss.

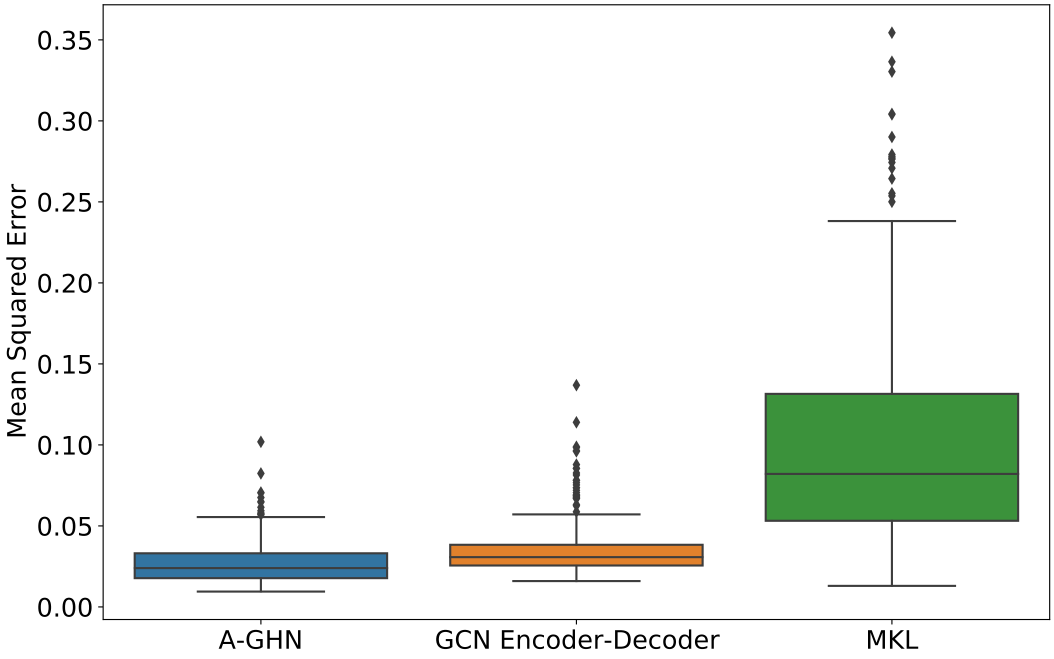

Figure SF2. Mean Square Error (MSE) values between empirical and predicted FCs of all the test subjects with the proposed A-GHN model (Green line), averaged over five runs, are compared with the predictions of the other two models. Horizontal lines show the mean MSE values (lower is better) of 0.0265, 0.037, and 0.086, respectively, for A-GHN, GCN Encoder-Decoder, and MKL.

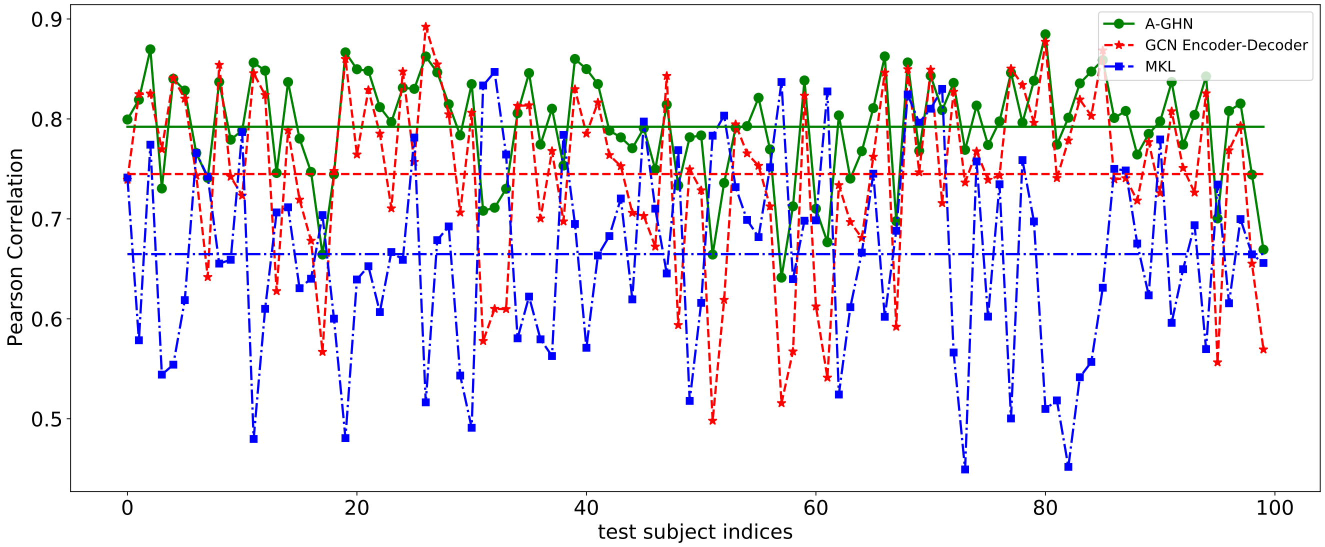

Figure SF3. Pearson correlation values between empirical and predicted FCs of all the test subjects with the proposed A-GHN model (Green line), averaged over five runs, are compared with the predictions of the other two models. Horizontal lines show the mean correlation values (higher is better) of 0.788, 0.732, and 0.661, respectively, for A-GHN, GCN Encoder-Decoder, and MKL.

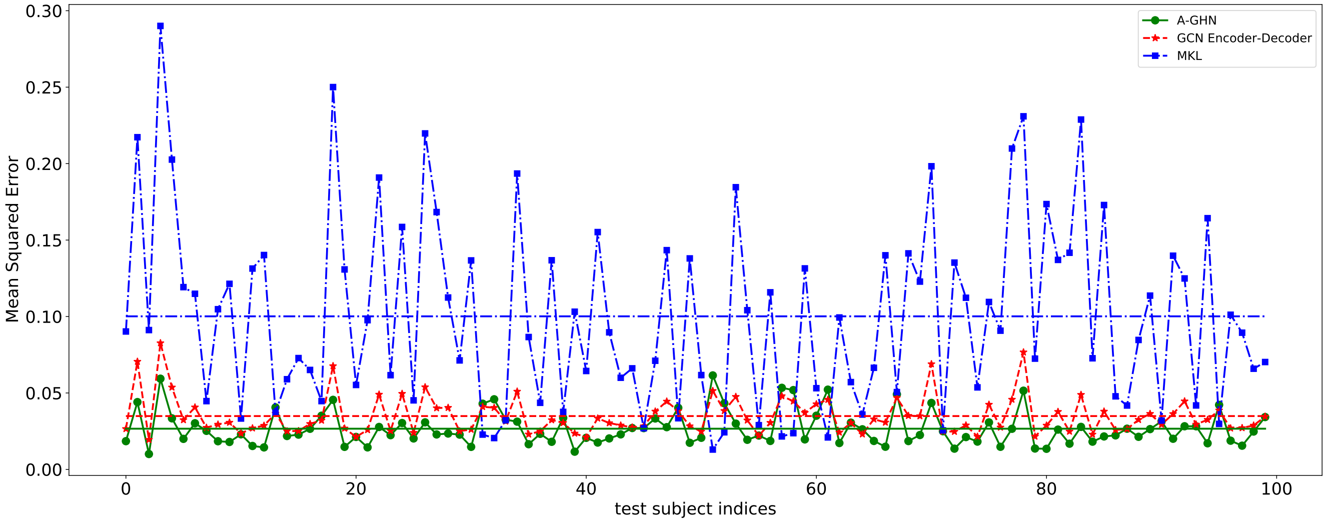

Figure SF4. Mean Square Error (MSE) values between empirical and predicted FCs of all the test subjects with the proposed A-GHN model (Green line), averaged over five runs, are compared with the predictions of the other two models. Horizontal lines show the mean MSE values (lower is better) of 0.0265, 0.037, and 0.086, respectively, for A-GHN, GCN Encoder-Decoder, and MKL.

1.1 Ablation Studies

Random A-GHN: In the Random A-GHN model, all the GHN models are kept frozen, and their corresponding biases and weight matrices are initialized uniformly at random. Hence, the attention module parameters are only trainable, and the remaining parameters are non-trainable in the Random A-GHN model. The results of the box-plot report Pearson correlation and MSE values for all 508 test subjects, as shown in Figures SF12 and SF5, respectively. Results point out that the overall Pearson and MSE values are better for A-GHN compared to Random A-GHN.

Varying the Training Data Size: Figure SF10 shows the mean Pearson correlations of the A-GHN model with varying training data set sizes. We ran the model with three different settings - 25%, 50%, and 75% of the data for training and subsequently tested it with the remaining data. As expected, the mean correlation of the proposed model increases with the size of the training

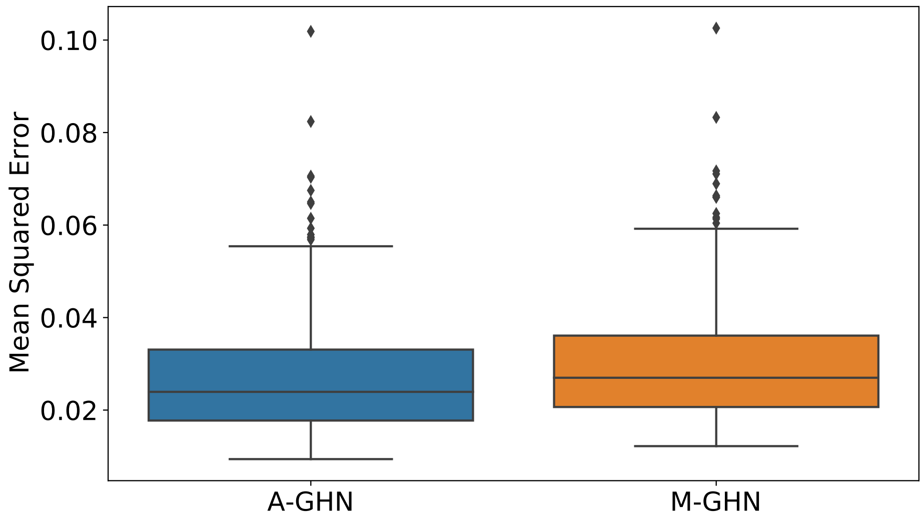

Figure SF5. Results of MSE (lower the better) comparison between A-GHN (with Attention) vs. M-GHN (Multiple GHNs, without Attention) on the test subjects, yielding better performance for the former model.

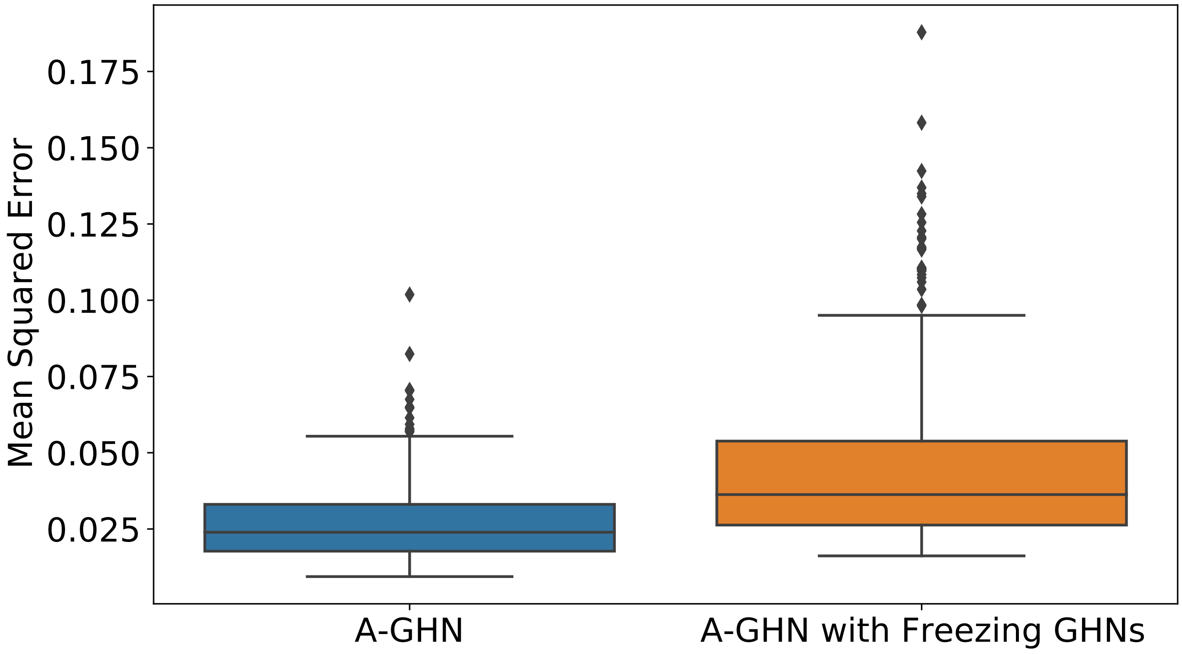

Figure SF6. Results of MSE (lower the better) comparison between A-GHN (full training) vs. A-GHN (Frozen/randomly-initialized GHNs) on the test subjects, indicating superior performance of the former model.

set – from 0.776 (25%), 0.788 (50%) to 0.79 (75%). However, the increase in performance is marginal as the A-GHN model yields a similar level of performance with a smaller training dataset, possibly because the graph convolutional network captures essential characteristics of the SC-FC mapping even with a sparse training set.

Perturbing the Model Input: In the second perturbation study, we trained the A-GHN model with perturbed SCs during the training phase and tested using the ground truth SC-FC pairs. Basically, this experiment tries to check the influence of training with broken training set on the target prediction ability. To assess whether FC prediction relies on the specific test SCs during the training phase, we considered the 550 random subjects used in the 5-run experiments reported earlier. Here, each training subject's SC matrix was perturbed $N = 100$ times by randomly generating the values of the elements from a power-law distribution that the elements are known to follow[1]. 100 perturbed sets of SCs for each training subject were used to

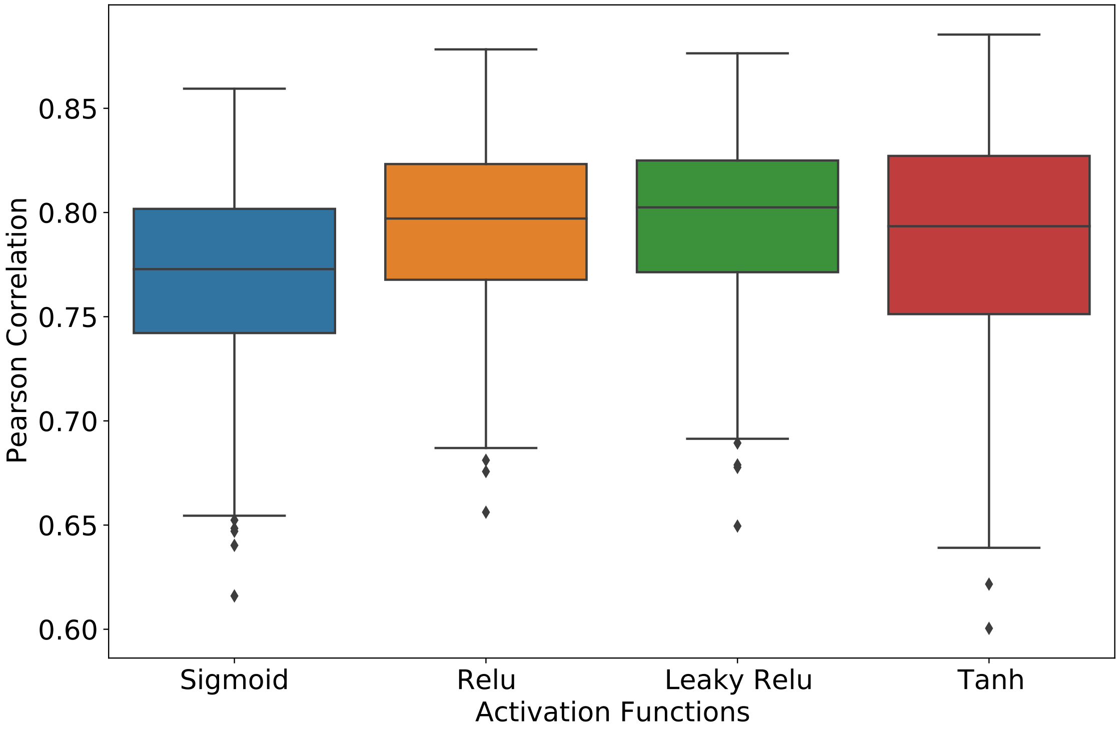

Figure SF7. Comparison of A-GHN performance on different activation functions at the output layer.

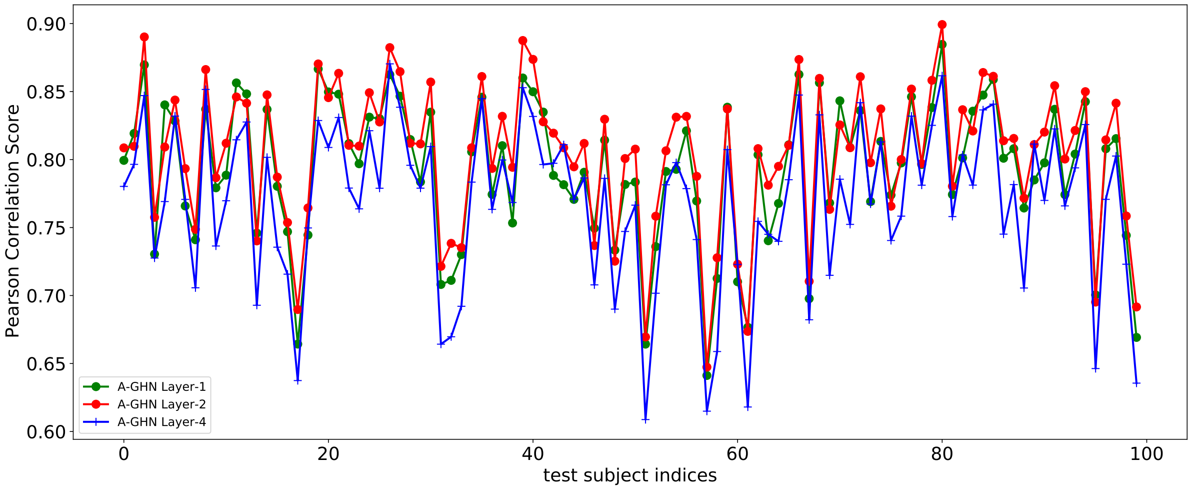

Figure SF8. Influence of increasing the number of hidden layers on the performance of the A-GHN model.

train independent A-GHN models. These trained models were used for testing with the correct test SC-FC pairs (508 subjects). Figure SF15 (please refer to the Supplementary) depicts the histogram of the mean performances across all the sets. The mean correlation is around 0.337 with the correlation values ranging in [0.08, 0.56]. The poor performance indicates the importance of training with meaningful structure-function relationships in order to yield valid predictions of the FC. An F-test establishes that the predictions from perturbed SCs and original SCs are statistically significantly different [$F(1,1014)=11089.4$, p=.000].

1.2 Leave-One-Out Results on 100 HCP subjects with AAL Atlas

LOOCV is conducted in a standard manner where the train-test splits comprise 99:1 participants. Of the 99 participants' data, 90 were used for training and 9 for validation. For ease of comparison, we depict the results of 98 participants in Figure SF16 (reported in the Supplementary).

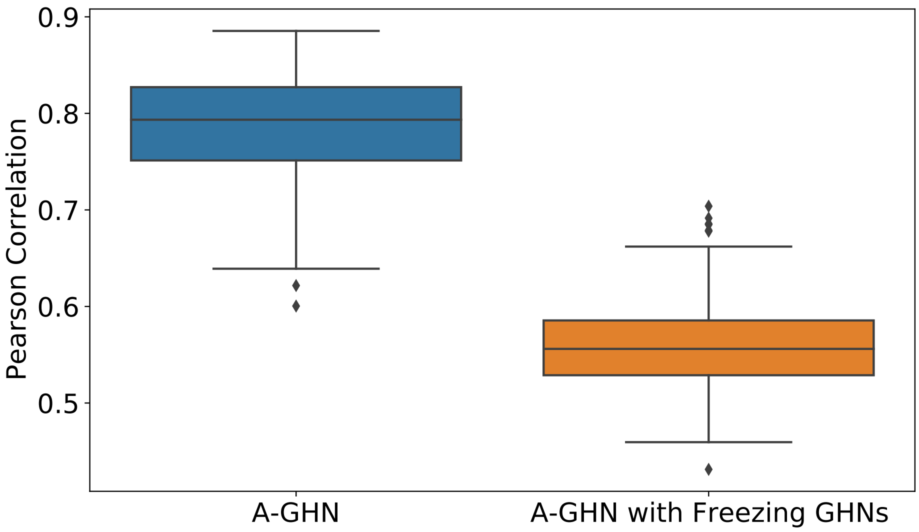

Figure SF9. Results of Pearson Correlation (higher the better) comparison between A-GHN (full training) vs. A-GHN (Frozen/randomly-initialized GHNs) on the test subjects.

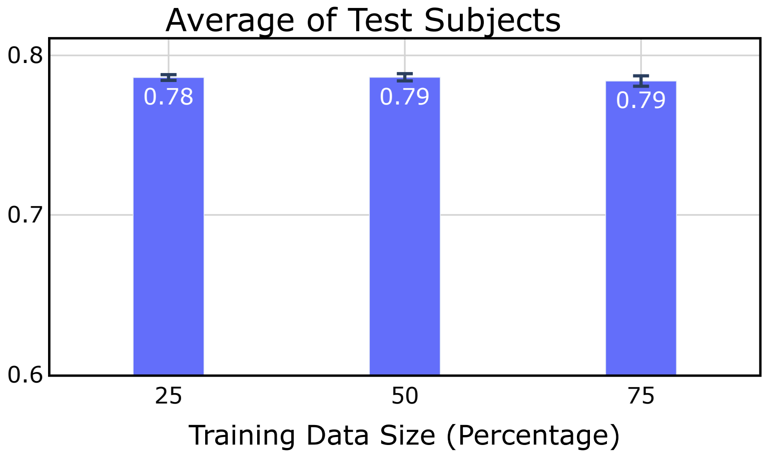

Figure SF10. Effect of changing the training set size on the model performance.

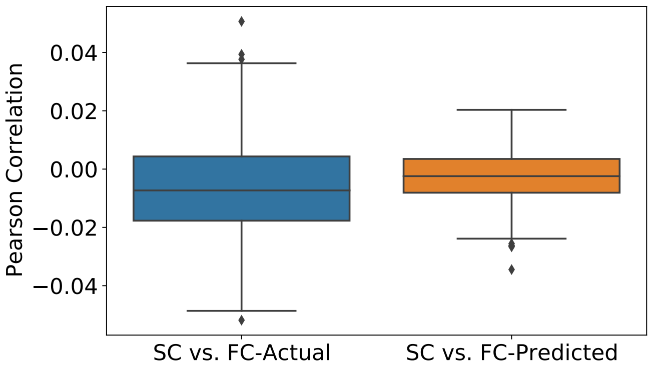

Figure SF11. Results of Pearson Correlation comparison between SC vs. FC (Ground Truth) (mean correlation: -0.0065) and SC vs. FC (Predicted) (mean correlation: -0.0024) on the test subjects, yielding a comparative performance.

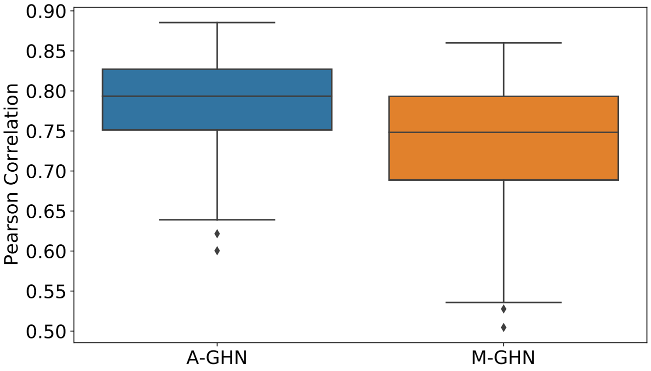

Figure SF12. Results of Pearson Correlation (higher the better) comparison between A-GHN (with Attention) vs. M-GHN (Multiple GHNs, without Attention) on the test subjects.

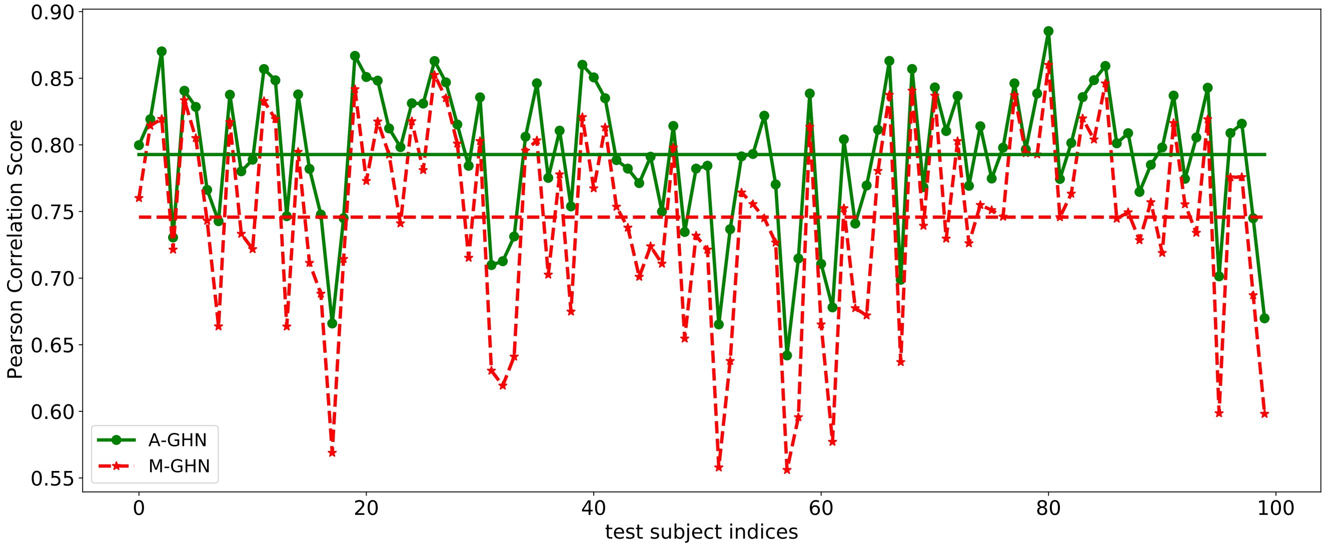

Figure SF13. Results of Pearson Correlation (higher the better) comparison between A-GHN (with Attention) vs. M-GHN (Multiple GHNs, without Attention) on the test subjects yield a comparative performance. Note that the subject indices are kept identical to those in Figure 4.

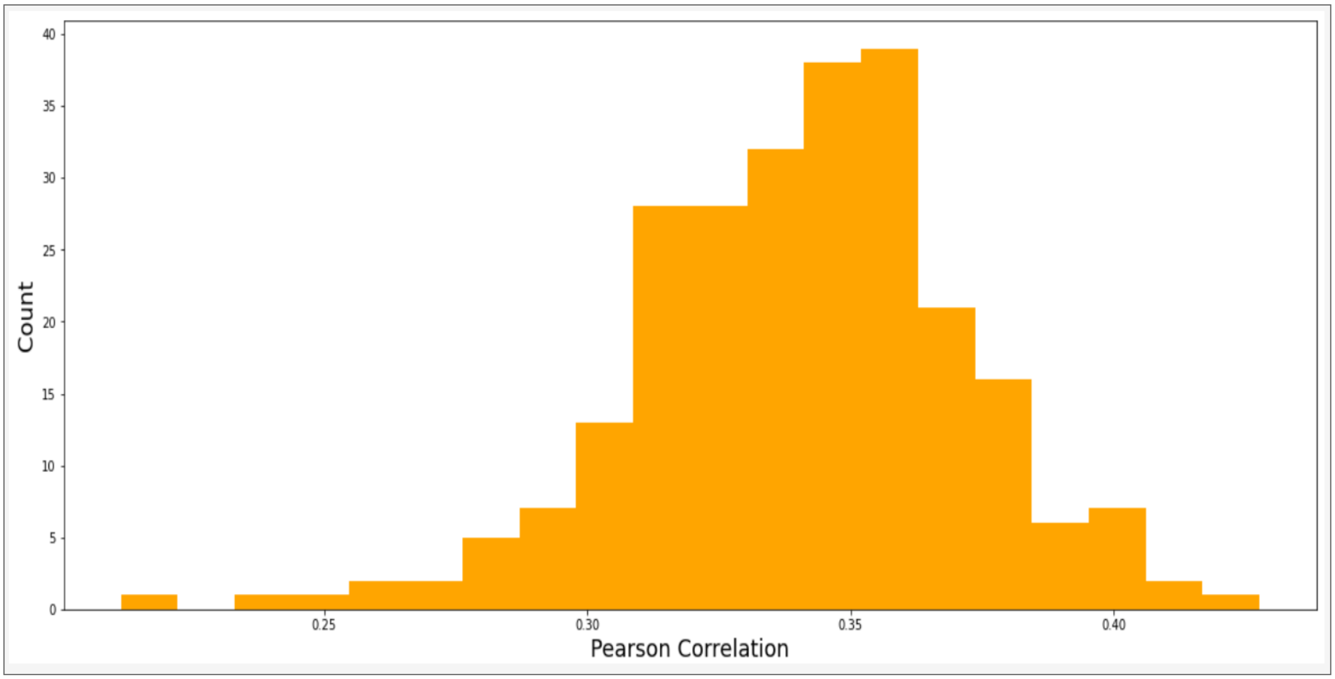

Figure SF14. Distribution of the average Pearson correlation scores in the perturbation experiments related to the testing dataset. The correlation scores between the predicted FC estimated using randomly perturbed SCs (N = 250 sets) of the test subjects and the ground truth FC are considered here. Randomizing the SC inputs for the test dataset seems to impair the performance severely.

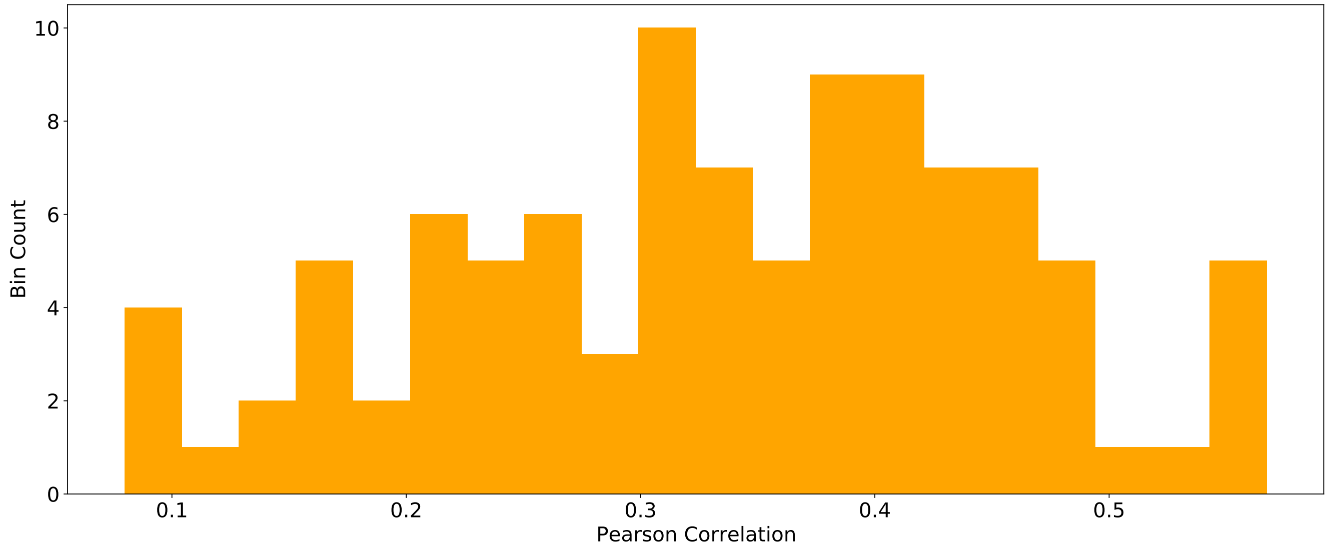

Figure SF15. Input perturbation while training the A-GHN model. The 100 perturbed sets of SCs were used to train independent A-GHN models. These trained models were used for testing with the correct test SC-FC pairs.

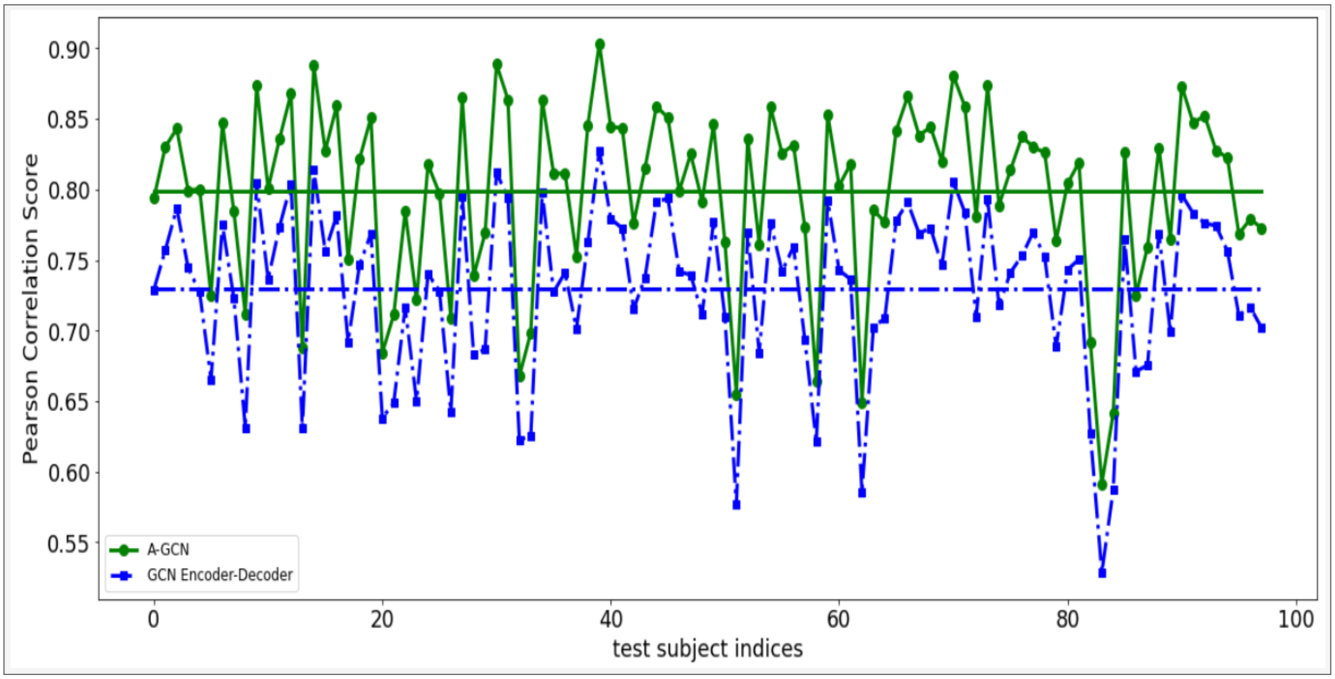

Figure SF16. 100 subjects AAL atlas: Pearson Correlation Results of leave-one-out cross-validation (LOO-CV) on the test subjects of A-GHN performs better than the other two models: GCN Encoder-Decoder and MKL. Note that the subject indices are kept identical to those in Figure 4.

2 Additional Results

We now perform our proposed A-GHN to other brain parcellations, including those with 114, 219, and 448 parcels based on the Lausanne anatomical atlas, as proposed in *Vázquez-Rodríguez et al. 2019*[4]. We use the publicly available data from[4], which comprises a total of 70 healthy participants. The dataset can be downloaded from following repository [`https://zenodo.org/records/2872624`].

Similar to supplementary Figure SF3, we repeat the similar experiments and report the results for randomly sampled 35 test subjects from the total 70 subjects in Figure SF22. Figure SF22 reports the performance of A-GHN model on various

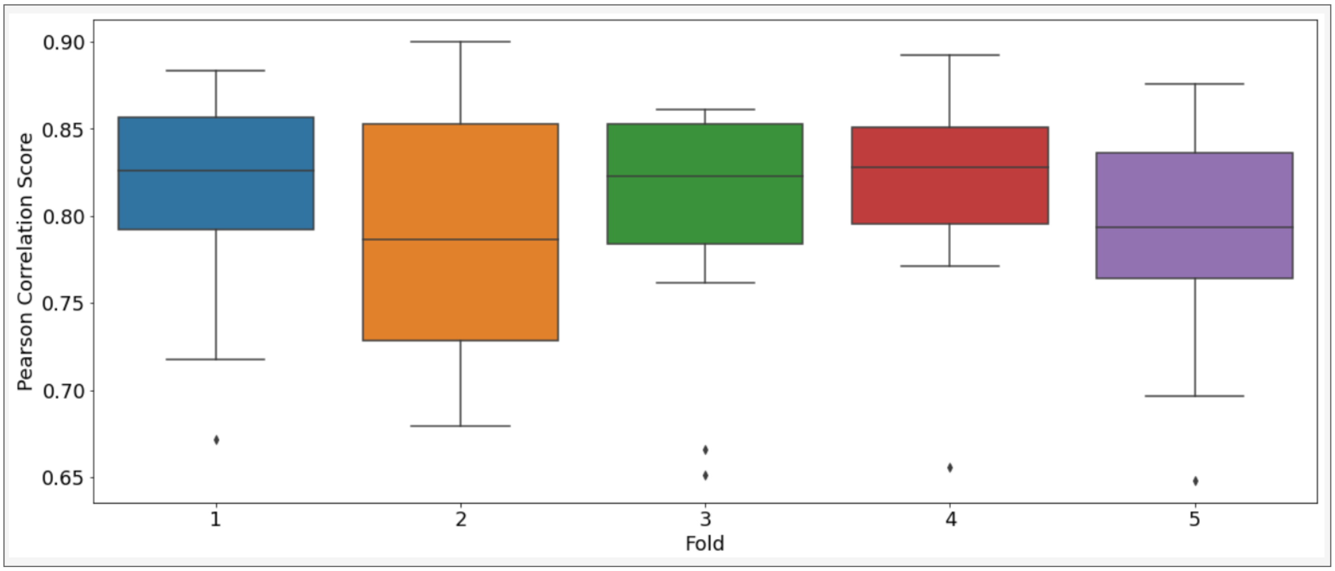

Figure SF17. Results of performance of A-GHN model in the 5-fold cross-validation setting on 100 subjects AAL atlas. The box plots depict the Pearson correlation between empirical and predicted FCs in each fold.

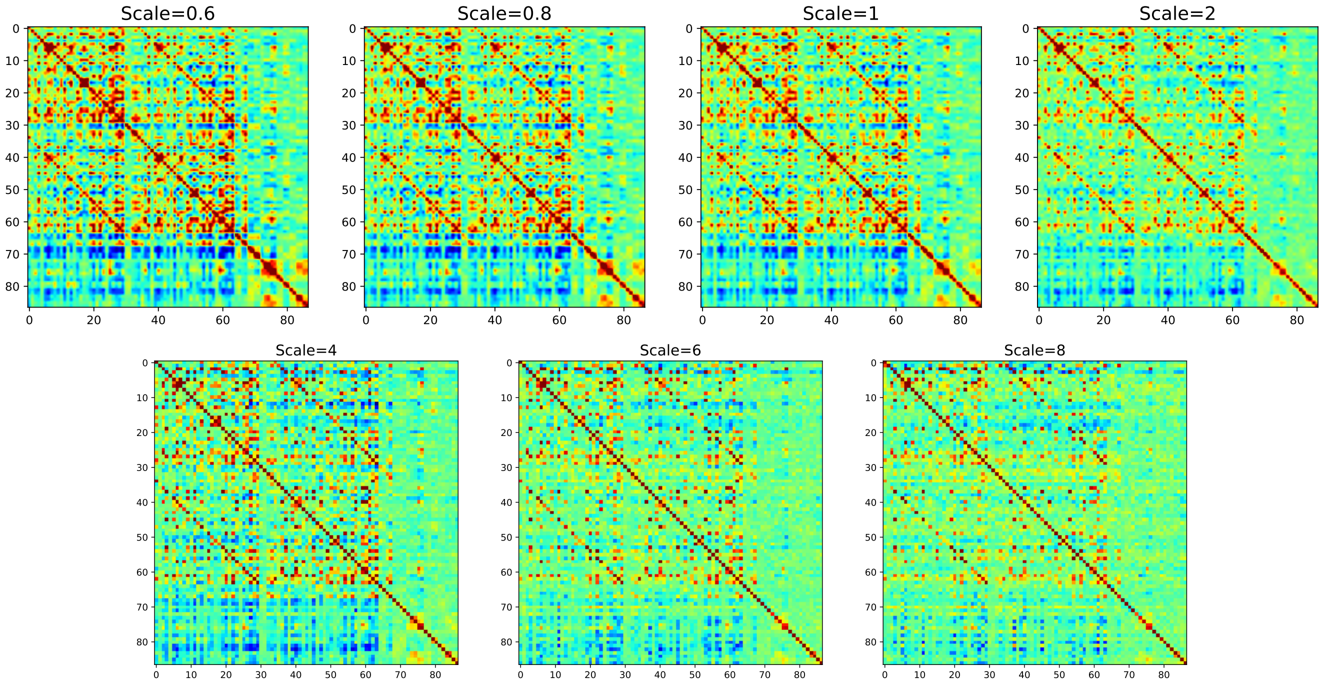

Figure SF18. Distinctness of learned weights (W_{γ_i}'s) corresponding to A-GHN sub-models. We check the distinctness of W_{γ_i}'s for every scale value ranging from i = 1, $\cdots$, m (= 7). Each of these m matrices is of 87×87 dimension.

parcellations. It can be observed from Figure SF22 that A-GHN model yield higher Pearson correlation information with ground truth FCs across all the test subjects, across three different parcellations: 0.638 (114-parcel), 0.586 (219-parcel) and 0.535 (448-parcel). The minimum and maximum Pearson correlation values across parcellations are as follows: (i) 114 - [0.571, 0.726], (ii) 219 - [0.541, 0.639], and (iii) 448 - [0.503, 0.567]. In summary, the experimental results across different parcellations consistently indicate that our proposed A-GHN model effectively captures the correlation structure of BOLD functional resting-state brain networks, as evidenced by Pearson correlations consistently exceeding 0.5 regardless of the chosen parcellation.

We compared the performance of our proposed model with the GCN-based Encoder-Decoder model. Figure SF23 reports

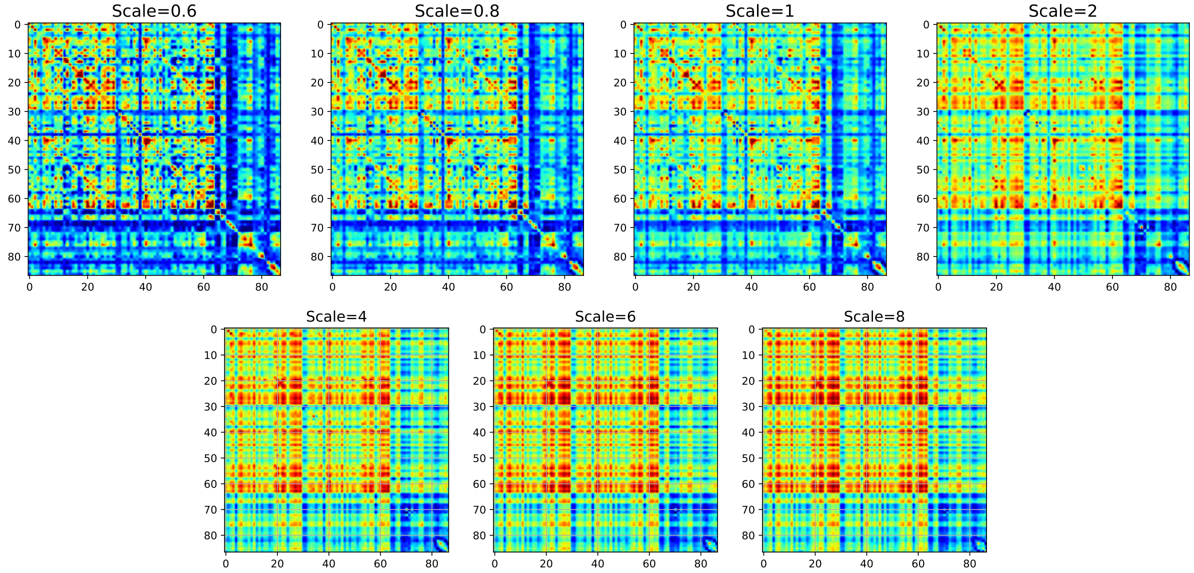

Figure SF19. Distinctness of ψ_{γ_i}'s. After scale-specific A-GHN sub-models are learned, we check the distinctness of ψ_{γ_i}'s for every scale value ranging from i = 1,···, m (= 7). Each of these m matrices is of 87×87 dimension.

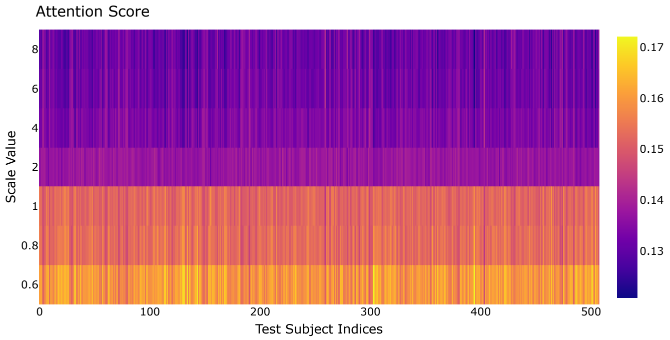

Figure SF20. The Figure showcase the contribution of the attention scores associated with each scale-specific A-GHN sub-model output related to a testing dataset in the A-GHN model. The attention score probabilities for the smaller scales are marginally higher than large-scale.

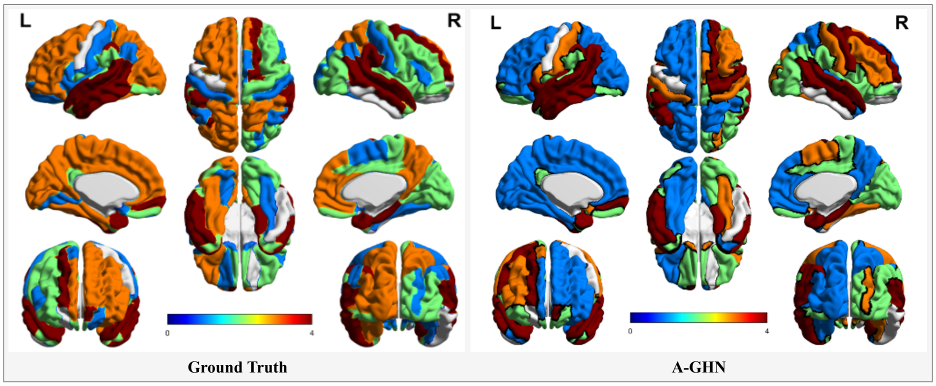

Figure SF21. The distinct, modular communities uncovered in empirical FCs (Ground Truth) and predicted FCs using the A-GHN model. The communities detected using BCT[2] are mapped onto the brain surface using BrainNet Viewer[3]. The colors are mapped according to the communities uncovered by the stochastic Louvain algorithm defined in BCT. Thus, the color scale cannot be directly compared between ground truth and A-GHN.

the performance of A-GHN and GCN-based Encoder-Decoder models on various parcellations. From Figure SF23, we can see that the proposed A-GHN model performs better with a mean correlation value of 0.638 (114-parcel), 0.586 (219-parcel) and 0.535 (448-parcel) on the test set as compared to GCN-based Encoder-Decoder model 0.572, 0.425 and 0.407.

We now computed the mean of the predicted FC and the mean of the empirical FC matrices of the test subjects across three different parcellations. The visualizations of FC matrices are shown in Figure SF24. Here, we can observe a better qualitative match between the mean predicted FC of our proposed model and the mean ground truth.

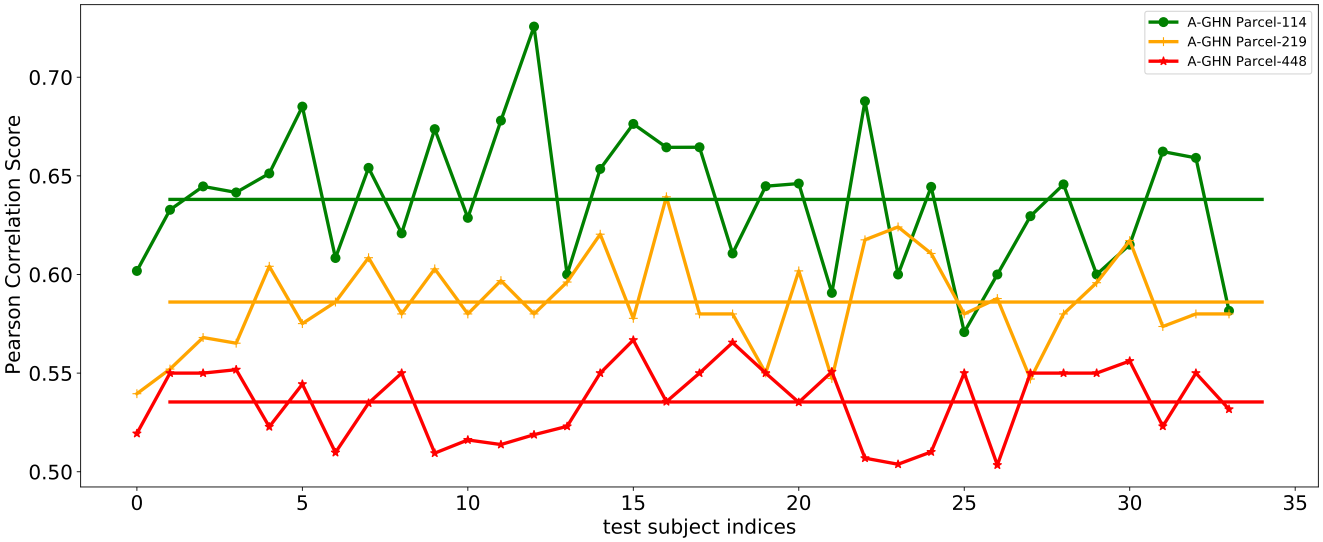

Figure SF22. Pearson correlation values between empirical and predicted FCs of all the test subjects with the proposed A-GHN model, averaged over five runs. Horizontal lines show the mean correlation values of 0.638, 0.586, and 0.535, respectively, for A-GHN across three different parcellations - 114, 219 and 448.

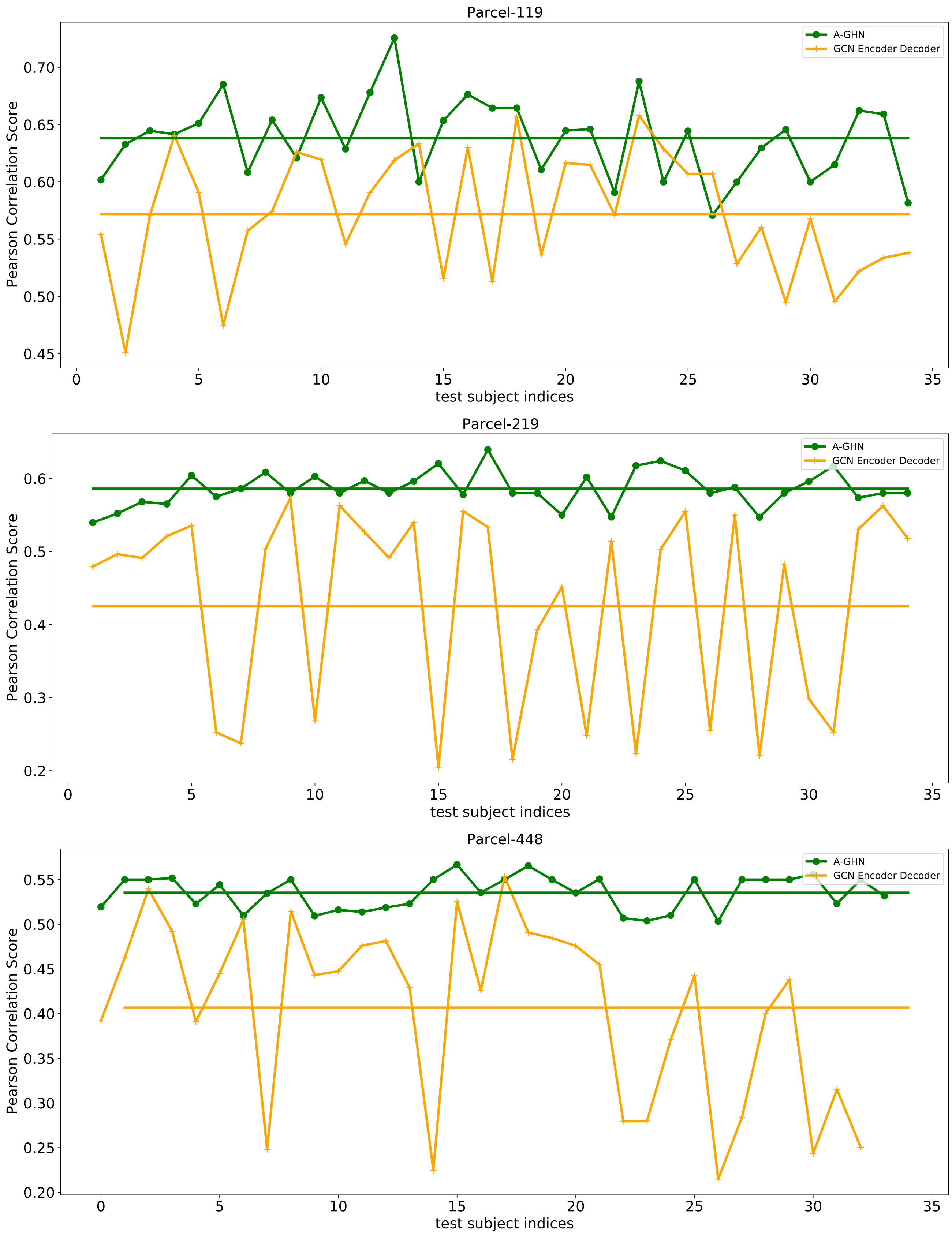

Figure SF23. Pearson correlation values between empirical and predicted FCs of all the test subjects with the proposed A-GHN model and GCN-based Encoder-Decoder model, averaged over five runs. Horizontal lines show the mean correlation values for A-GHN and GCN-based Encoder-Decoder models across three different parcellations - 114 (*Top row*), 219 (*Middle row*) and 448 (*Bottom row*).

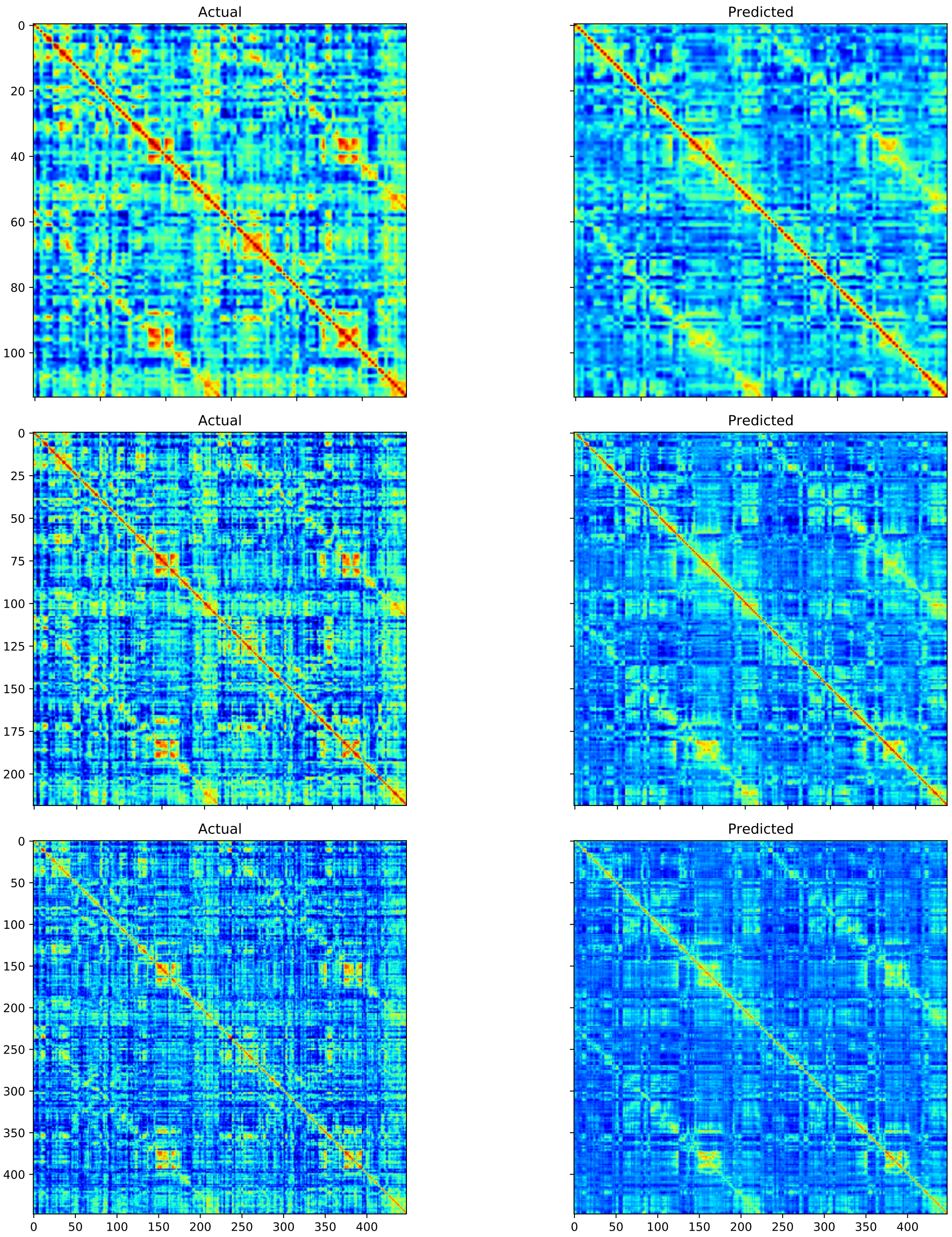

Figure SF24. Qualitative comparison of the Functional Connectivity matrices (FCs). The mean of the predicted FCs from the proposed A-GHN model is compared with that of the mean FC from ground truth (empirically observed), across three different parcellations, including, 114 (*Top row)*, 219 (*Middle row*) and 448 (*Bottom row*).

References

1. Clauset, A., Shalizi, C. R. & Newman, M. E. Power-law distributions in empirical data. *SIAM review* **51**, 661–703 (2009).
2. Rubinov, M. & Sporns, O. Complex network measures of brain connectivity: uses and interpretations. *Neuroimage* **52**, 1059–1069 (2010).
3. Xia, M., Wang, J. & He, Y. Brainnet viewer: a network visualization tool for human brain connectomics. *PloS one* **8**, e68910 (2013).
4. Vázquez-Rodríguez, B. *et al.* Gradients of structure–function tethering across neocortex. *Proc. Natl. Acad. Sci.* **116**, 21219–21227 (2019).
